# Driving pressure-guided ventilation during one-lung ventilation for thoracic surgery: a systematic review and meta-analysis

**DOI:** 10.3389/fmed.2026.1837064

**Published:** 2026-05-20

**Authors:** Xinrui Yin, Shijia Du

**Affiliations:** 1 Department of Anesthesiology, Aerospace Center Hospital, Beijing, China; 2 Department of VIP Dental Service, Peking University Stomatological Hospital, Beijing, China

**Keywords:** driving pressure, lung-protective ventilation, one-lung ventilation, postoperative pulmonary complications, thoracic anesthesia, thoracic surgery

## Abstract

**Background:**

Driving pressure-guided ventilation has been proposed as a physiologically rational lung-protective strategy during one-lung ventilation for thoracic surgery. However, no previous systematic review has specifically focused on trials in which driving pressure was the explicit primary ventilatory target during one-lung ventilation. We aimed to assess the effects of driving pressure-guided ventilation on postoperative pulmonary complications (PPCs) and related perioperative outcomes in adults undergoing thoracic surgery.

**Methods:**

We searched MEDLINE, Embase, CENTRAL, Web of Science, Scopus, and trial registries from inception to 19 March 2026. We included randomized controlled trials comparing explicit driving pressure-guided ventilation with conventional non-driving-pressure-guided ventilation during one-lung ventilation in adults undergoing thoracic surgery. Observational studies were summarised narratively. Random-effects meta-analyses were performed using restricted maximum likelihood estimation with Hartung–Knapp adjustment. Certainty of evidence was assessed using GRADE. The review was registered in PROSPERO (CRD420261329253).

**Results:**

Four randomized controlled trials (654 patients) were included in the primary analysis. The pooled effect on PPCs was not statistically significant in the primary random-effects analysis (risk ratio [RR]: 0.60, 95% confidence interval [CI]: 0.26–1.35; *p* = 0.14; *I*^2^ = 49.7%). Exploratory supportive analyses—including a fixed-effect model (RR 0.61; *p* = 0.007), the pooled absolute risk difference (−7.6%; *p* = 0.027), and a broadened sensitivity analysis of six trials incorporating individualised PEEP strategies (RR 0.70; *p* = 0.12)—were directionally concordant but should not be interpreted as independent confirmation of effect, given the imprecision of the primary estimate and variation in baseline PPC risk. Four observational studies provided mixed supplementary evidence. The overall certainty of evidence was low according to GRADE, owing to inconsistency and imprecision.

**Conclusion:**

The primary randomized evidence did not demonstrate a statistically significant reduction in postoperative pulmonary complications, and the overall certainty of evidence was low. The available data are therefore hypothesis-generating. Larger, multicentre, and geographically diverse randomized trials with harmonised outcome definitions are needed to clarify whether driving pressure-guided ventilation improves postoperative outcomes and should be adopted more broadly in thoracic anaesthesia.

**Systematic review registration:**

https://www.crd.york.ac.uk/PROSPERO/view/CRD420261329253, Unique Identifier: CRD420261329253.

## Introduction

One-lung ventilation is a fundamental component of thoracic anaesthesia, enabling surgical access to the non-dependent hemithorax during lung resection, oesophagectomy, and other intrathoracic procedures (1). However, ventilation of a single lung substantially reduces the available gas-exchanging volume and functional residual capacity, concentrating mechanical forces on the dependent lung and increasing the risk of ventilator-induced lung injury (1, 2). Postoperative pulmonary complications remain among the most common and clinically consequential adverse events after thoracic surgery, contributing to prolonged hospital stay, increased healthcare costs, and perioperative mortality (2, 3). Conventional lung-protective ventilation strategies during one-lung ventilation have traditionally relied on fixed low tidal volumes and moderate positive end-expiratory pressure, yet these approaches do not account for individual variation in respiratory system compliance (4). Driving pressure—defined as plateau pressure minus positive end-expiratory pressure—has emerged as a physiologically attractive target for intraoperative ventilator management because it reflects the mechanical burden imposed on aerated lung tissue relative to the available functional lung volume, rather than relying on absolute ventilatory settings alone (5).

Several randomized controlled trials and observational studies have examined the relationship between intraoperative driving pressure and postoperative outcomes during one-lung ventilation (6, 7). However, the evidence base remains limited and fragmented. Individual trials have been small, mostly single-centre, and heterogeneous in intervention design, comparator strategy, and outcome definition. Some trials have targeted driving pressure minimisation through positive end-expiratory pressure titration, whereas others have used tidal-volume adjustment to achieve a predefined driving pressure range, raising the question of whether different implementation pathways yield equivalent clinical effects. To our knowledge, no previous systematic review has specifically focused on trials in which driving pressure was the explicit primary ventilatory target during one-lung ventilation, nor has any prior synthesis clearly separated strict driving pressure-guided evidence from the broader literature on individualised ventilation strategies (8).

This systematic review and meta-analysis aimed to evaluate whether driving pressure-guided ventilation, compared with conventional non-driving-pressure-guided ventilation, reduces postoperative pulmonary complications and improves related perioperative outcomes in adults undergoing thoracic surgery with one-lung ventilation. We applied a two-layer evidence synthesis framework in which randomized controlled trials formed the primary quantitative analysis and observational studies were synthesised narratively as supplementary evidence, thereby preserving the distinction between interventional evidence from trials and associative evidence from observational studies. This approach provides a focused and methodologically transparent assessment of the current evidence base for driving pressure-guided ventilation in thoracic anaesthesia.

## Methods

### Protocol registration and reporting

This systematic review and meta-analysis was prospectively registered in PROSPERO (CRD420261329253; registered 1 March 2026). The review was conducted and reported in accordance with the Preferred Reporting Items for Systematic Reviews and Meta-Analyses (PRISMA) 2020 statement (9). Both postoperative pulmonary complications (PPCs) and clinically significant intraoperative haemodynamic adverse events were prespecified as co-primary outcomes in the registered PROSPERO protocol. During data extraction, we identified that none of the included randomized trials reported intraoperative haemodynamic adverse events as a defined safety endpoint. Although several trials reported intraoperative vasopressor use, these data were recorded as descriptive intraoperative characteristics with substantial definitional heterogeneity, ranging from rescue-only ephedrine use to overall prophylactic plus rescue vasopressor use. Before quantitative synthesis, this outcome was therefore reclassified as an exploratory secondary outcome (intraoperative vasopressor use). This deviation from the registered protocol is reported transparently in accordance with PRISMA 2020 guidance. As a consequence, only one of the two originally registered co-primary outcomes—postoperative pulmonary complications—could ultimately be assessed as planned.

### Eligibility criteria

Eligibility criteria were defined *a priori* using the Population–Intervention–Comparator–Outcomes–Study design (PICOS) framework.

#### Population

We included adults (≥18 years) undergoing elective thoracic surgery requiring one-lung ventilation (OLV) under general anaesthesia with mechanical ventilation. Eligible procedures included lung resection, oesophagectomy, mediastinal surgery, and related thoracic operations performed via video-assisted thoracoscopic surgery (VATS) or open approaches. No restrictions were applied regarding baseline comorbidities or ASA physical status.

#### Intervention (driving pressure–guided ventilation)

We included any intraoperative ventilation strategy in which driving pressure [ΔP = plateau pressure (Pplat) – PEEP] was explicitly used as the primary target for ventilator adjustment during OLV—either by minimising ΔP or by maintaining ΔP below a prespecified threshold (e.g., ≤14 or ≤15 cm H₂O), as clearly stated by the original investigators. Strategies could achieve the ΔP target via tidal-volume reduction, PEEP titration, combined optimisation, or algorithm-driven protocols. A key eligibility requirement was that the study explicitly described ΔP minimisation or threshold attainment as the principal decision rule for ventilator settings during OLV.

##### Operational classification of explicitly driving pressure-guided ventilation

To ensure reproducible classification, trials were classified as explicitly driving pressure-guided when the published report clearly identified driving pressure as the principal ventilatory target or decision rule during OLV, and when the intervention protocol operationalised this target either by minimisation of driving pressure or by maintenance below a predefined threshold. Trials in which lower driving pressure arose as a downstream consequence of another primary target—for example, PEEP titration to maximum compliance or electrical impedance tomography-guided PEEP selection—were not classified as strict DP-guided trials, and were instead retained for the broadened sensitivity analysis described below. Classification was performed independently by two reviewers after full-text eligibility assessment, using verbatim-extracted intervention statements, with disagreements resolved through discussion and third-reviewer adjudication where needed. For each classified trial, the verbatim intervention statement, the classification outcome, and the rationale are provided in Supplementary Table S1.

#### Comparator

Comparators were conventional or non–ΔP-targeted ventilation strategies in which ventilator adjustments were not primarily guided by driving pressure, including fixed tidal volume/PEEP approaches, clinician judgement–guided ventilation, or strategies primarily targeting other parameters (e.g., oxygenation) without ΔP being the primary adjustment target.

#### Outcomes

Studies were eligible if they reported at least one extractable outcome of interest, including postoperative pulmonary complications (PPCs), intraoperative haemodynamic adverse events, oxygenation indices, ventilatory mechanics, resource use outcomes, or mortality, as prespecified. We anticipated that between-study heterogeneity in the operational definition of PPCs and in the postoperative ascertainment window would be substantial, reflecting the absence of a universal PPC definition in thoracic anaesthesia research. We therefore preferentially extracted the study-reported overall or composite PPC outcome as defined by the original investigators, and did not attempt to harmonise or reconstruct a common PPC definition across trials, because the component-level data required for harmonisation were not consistently reported. Outcome-level heterogeneity introduced by varying PPC definitions is explicitly acknowledged as a source of between-study variability that could not be eliminated.

#### Study design and evidence layers

We applied a two-layer synthesis framework. The primary analyses included randomised controlled trials (parallel-group or crossover designs). Observational studies (prospective or retrospective cohort studies) were eligible as supplementary evidence. Because the identified observational studies assessed intraoperative driving pressure as an exposure variable rather than comparing a DP-guided strategy with a conventional ventilation strategy, they were synthesised narratively rather than pooled quantitatively, and were not combined with RCTs.

#### Exclusion criteria

We excluded studies enrolling paediatric patients (<18 years), studies in which OLV was not the primary ventilation context (e.g., cardiac surgery), case reports, reviews or editorials, and conference abstracts without sufficient data for extraction. We also excluded studies that altered multiple ventilation parameters without clearly stating ΔP as the primary guiding target, and studies in which driving pressure was not reported or could not be derived with available data.

### Search strategy and study selection

#### Search strategy

We systematically searched the following electronic databases from inception to 19 March 2026: MEDLINE (via PubMed), Embase (via Embase.com), the Cochrane Central Register of Controlled Trials (CENTRAL), Web of Science Core Collection, and Scopus. To identify ongoing or unpublished studies, we also searched ClinicalTrials.gov and the World Health Organization International Clinical Trials Registry Platform (WHO ICTRP). In addition, we manually screened reference lists of included studies and relevant reviews to identify potentially eligible articles not captured by database searches.

The search strategy combined controlled vocabulary (e.g., MeSH and Emtree terms) and free-text keywords related to one-lung ventilation, driving pressure (ΔP) and its components (plateau pressure, PEEP, respiratory system compliance), and thoracic surgical procedures. No restrictions were applied regarding language, country, or publication date. The full search strategies for all databases are provided in Supplementary Appendix S1.

#### Study selection

All records retrieved from the searches were imported into a reference-management software for de-duplication. Two reviewers independently screened titles and abstracts for potential eligibility. Full texts were subsequently assessed independently by the same reviewers against the prespecified eligibility criteria. Disagreements at any stage were resolved through discussion and consensus; if consensus could not be reached, a third reviewer adjudicated.

For each study included after full-text review, the study design was classified as an RCT (primary evidence layer) or an observational cohort study (supplementary evidence layer), which determined its placement in the corresponding synthesis framework. Reasons for exclusion at the full-text stage were recorded, and the study selection process was documented using a PRISMA 2020 flow diagram.

### Data extraction and outcomes

#### Data extraction

Data were extracted using a piloted, standardised data-collection form. Two reviewers independently extracted data from each included study, cross-checked all entries, and resolved discrepancies by discussion; unresolved disagreements were adjudicated by a third reviewer. When multiple reports described the same study population, we collated information across reports and extracted outcomes from the most complete dataset, avoiding double counting.

We extracted study characteristics (first author, year, country, design, trial registration, sample size, funding, and conflicts of interest), participant and surgical characteristics (age, sex, body mass index, ASA physical status, pulmonary risk indicators where available, procedure type, and surgical approach), and intervention/comparator details during OLV (ventilation mode, FiO₂, and any recruitment manoeuvre protocol).

For the intervention arm, we extracted the driving-pressure (ΔP) target definition (threshold or minimisation strategy) and the optimisation mechanism (predominantly tidal-volume–driven, predominantly PEEP-driven, or combined). To enhance reproducibility and minimise misclassification, the ΔP-target statement and decision rules were extracted verbatim (word-for-word) from the original report whenever possible. To verify that the intervention produced a clinically meaningful contrast, we extracted achieved intraoperative ΔP and related mechanics (ΔP, Pplat, PEEP, Vt, and respiratory system compliance) for both study arms at reported timepoints during OLV. When multiple intraoperative timepoints were reported, we prioritised values measured during a steady-state OLV period and extracted the timepoint closest to approximately 30 min after initiation of OLV; if unavailable, we used the closest to 60 min, and otherwise the most comparable prespecified intraoperative timepoint. The achieved between-group ΔP separation (intervention minus comparator) was calculated from extracted summary statistics and was used in prespecified sensitivity analyses (e.g., restricting to studies with an absolute ΔP difference ≥3 cmH₂O).

For dichotomous outcomes, we extracted event counts and totals per group (n/N), together with outcome definitions and assessment windows. For continuous outcomes, we extracted means and standard deviations (or standard errors/confidence intervals where convertible) per group, along with measurement timepoints and units. When outcomes were reported as medians with interquartile ranges, we recorded the original non-parametric summaries and flagged them for prespecified transformation methods (10, 11). Where required data were missing or unclear, we attempted to derive values from other reported statistics or contacted study authors when feasible.

#### Outcomes and definitions

Outcomes were prespecified and extracted according to the study-defined follow-up window (in-hospital or up to 30 days when reported). The co-primary outcomes were: (1) postoperative pulmonary complications (PPCs) and (2) clinically significant intraoperative haemodynamic adverse events. As documented in the Protocol registration and reporting section, the second co-primary outcome was reclassified as an exploratory secondary outcome before quantitative synthesis because of inconsistent reporting across included trials.

PPCs were defined as a composite outcome according to each study’s original definition. Where available, we preferentially extracted the study-reported overall/composite PPC outcome (including EPCO-aligned composites when explicitly used). We did not construct a *de novo* PPC composite by aggregating individual complications when an overall PPC definition was not provided; in such cases, individual complications were analysed separately as secondary outcomes. Prespecified PPC components included pneumonia, atelectasis, acute respiratory distress syndrome, respiratory failure, reintubation, prolonged mechanical ventilation, and other clinically relevant pulmonary events as defined by the original investigators.

Haemodynamic adverse events were defined as study-reported clinically significant hypotension or haemodynamic instability requiring vasoactive support (e.g., vasopressor initiation or escalation) during one-lung ventilation or intraoperatively, as per each study’s definition. When multiple haemodynamic endpoints were reported, we prioritised the endpoint most closely reflecting vasoactive requirement rather than isolated blood pressure thresholds.

Key secondary outcomes included: (1) oxygenation, assessed as PaO₂/FiO₂ at prespecified intraoperative timepoints during one-lung ventilation (prioritising approximately 30 and 60 min when available) and the need for hypoxaemia rescue interventions; (2) ventilatory mechanics and implementation outcomes to verify intervention fidelity, including achieved driving pressure (ΔP), plateau pressure (Pplat), PEEP, tidal volume (Vt, preferably indexed to predicted body weight), and respiratory system compliance (Crs); and (3) resource use and clinical outcomes, including ICU admission, ICU length of stay, hospital length of stay, and in-hospital or 30-day mortality.

When outcomes were reported at multiple timepoints, we extracted the most clinically comparable prespecified timepoint across studies, prioritising one-lung ventilation steady-state periods where feasible, and recorded all available timepoints for descriptive synthesis and sensitivity analyses.

### Risk of bias assessment and certainty of evidence

#### Risk of bias assessment

Two reviewers independently assessed risk of bias for each included study using design-appropriate tools, with disagreements resolved by consensus or adjudication by a third reviewer.

For RCTs, risk of bias was assessed using the Cochrane Risk of Bias 2 (RoB 2) tool across five domains (12): (1) bias arising from the randomisation process, (2) bias due to deviations from intended interventions, (3) bias due to missing outcome data, (4) bias in measurement of the outcome, and (5) bias in selection of the reported result. Each domain was judged as “low risk,” “some concerns,” or “high risk,” and an overall RoB 2 judgement was assigned according to the RoB 2 algorithm, prioritising the co-primary outcomes. Detailed domain-level judgements for each RCT are presented in Supplementary Table S2.

For observational cohort studies, risk of bias was assessed using the Risk Of Bias In Non-randomized Studies of Interventions (ROBINS-I) tool (13), evaluating bias due to (1) confounding, (2) selection of participants, (3) classification of interventions, (4) deviations from intended interventions, (5) missing data, (6) measurement of outcomes, and (7) selection of the reported result. Each domain was judged as “low,” “moderate,” “serious,” or “critical” risk of bias, leading to an overall ROBINS-I judgement. ROBINS-I was pre-specified in the PROSPERO protocol as the risk-of-bias framework for observational evidence. Although the included observational studies evaluate driving pressure as an exposure variable rather than as an allocated intervention, ROBINS-I was retained because (i) it was pre-specified at the protocol stage, (ii) its seven domains systematically address confounding—the principal methodological concern in exposure–outcome studies of driving pressure, and (iii) the intervention/exposure domain can be adapted to an exposure contrast (higher vs. lower driving pressure). To complement this pre-specified assessment, we additionally performed a post-hoc appraisal using the QUality In Prognosis Studies (QUIPS) tool (14), which is specifically designed for prognostic and exposure research. The ROBINS-I assessment is presented in Supplementary Table S3, and the QUIPS appraisal is presented in Supplementary Table S4. In keeping with our two-layer synthesis framework, observational risk-of-bias assessments were used to appraise the quality of supplementary observational evidence and to inform its narrative interpretation; they were not combined with RCT RoB 2 judgements.

#### Certainty of evidence

We assessed the certainty of evidence for the co-primary outcomes in the RCT evidence layer only using the Grading of Recommendations Assessment, Development and Evaluation (GRADE) approach (15). Certainty was rated as high, moderate, low, or very low based on risk of bias, inconsistency, indirectness, imprecision, and publication bias. Evidence from RCTs began at “high” certainty and was downgraded as appropriate according to GRADE guidance. Summary of Findings tables were produced for the co-primary outcomes based on RCT data. Observational evidence was synthesised and presented as supplementary, hypothesis-supporting information and was not incorporated into the primary GRADE ratings or Summary of Findings tables.

### Statistical analysis, subgroup and sensitivity analyses

#### Data synthesis and statistical analysis

We used a two-layer evidence synthesis framework. The primary (main) analyses included randomised controlled trials (RCTs) only, providing the highest level of causal inference. Observational studies were synthesised narratively as supplementary evidence owing to heterogeneity in study design (exposure–outcome association studies rather than comparative intervention designs) and were not pooled with RCTs.

Although all four primary trials satisfied the operational criteria for explicit driving pressure-guided ventilation (see Supplementary material), the strategies employed to achieve the driving pressure target differed mechanistically, with three trials using PEEP titration and one using tidal-volume titration. These pathways act on different physiological mechanisms and should not be assumed to be clinically equivalent. The pooled estimate should therefore be interpreted as a summary across a mechanistically heterogeneous intervention class, and the results are interpreted with appropriate caution.

For dichotomous outcomes, we used risk ratios (RRs) with 95% confidence intervals (CIs) as the primary effect measure, calculated from 2 × 2 tables where possible. Odds ratios (ORs) were explored in sensitivity analyses. For continuous outcomes, we used mean differences (MDs) when outcomes were reported on the same scale and units, and standardised mean differences (SMDs) when scales differed.

We performed random-effects meta-analyses using restricted maximum likelihood (REML) estimation with Hartung–Knapp adjustment for uncertainty in the between-study variance estimate. Statistical heterogeneity was assessed using Cochran’s *Q* and the *I*^2^ statistic, with *I*^2^ > 50% considered indicative of substantial heterogeneity. Where sufficient studies were available (typically ≥10), we planned exploratory meta-regression to investigate potential sources of heterogeneity.

For rare dichotomous outcomes with sparse data, we used the Mantel–Haenszel (MH) method as the primary pooling approach, which naturally accommodates single-zero-cell studies without requiring continuity corrections. We performed sensitivity analyses using Peto’s odds ratio when event rates were very low and treatment effects were not extreme. Trials with zero events in both arms were excluded from relative effect estimation (RR/OR); however, we conducted a prespecified sensitivity analysis using the risk difference (RD) to retain these trials and more fully characterise the safety profile. For single-zero-cell studies, we additionally assessed robustness by comparing results with and without continuity correction and across alternative rare-event methods.

When continuous outcomes were reported as medians with interquartile ranges, we estimated means and standard deviations using established methods (10, 11). Prior to conversion, we assessed the likelihood of skewness based on available distributional information and summary statistics; where substantial skewness was suspected, we explored the impact via sensitivity analyses excluding converted data.

Small-study effects and publication bias were assessed using funnel plots and Egger’s regression test when at least 10 studies were available for a given meta-analysis; otherwise, we stated that formal assessment was not reliable due to limited study numbers.

All statistical analyses were performed using R version 4.4.0 (R Foundation for Statistical Computing, Vienna, Austria) with the ‘meta’ (version 7.0–0) and ‘metafor’ (version 4.6–0) packages.

#### Subgroup analyses

Prespecified subgroup analyses (primarily within the RCT layer when data permitted) were planned to explore clinically plausible effect modifiers and sources of heterogeneity: DP-guided strategy type (predominantly tidal-volume–driven, predominantly PEEP-driven, or combined Vt + PEEP optimisation—classified according to the stated protocol and principal mechanism described by the original investigators), DP target threshold (ΔP ≤ 14 cmH₂O vs. ≤ 15 cmH₂O vs. other predefined targets/minimisation approaches), recruitment manoeuvres (routine RM use vs. no routine RM), surgical category (lung resection vs. oesophagectomy vs. mixed thoracic procedures), background tidal volume strategy (baseline/target Vt ≤ 6 mL/kg predicted body weight vs. > 6 mL/kg), and high-risk lung populations (COPD/reduced FEV₁ or other predefined high-risk subgroups), where sufficient data were available. A complete listing of all prespecified subgroup and sensitivity analyses, together with whether each was actually estimable given the final included evidence base, is provided in the Supplementary material to enhance methodological transparency.

#### Sensitivity analyses

Prespecified sensitivity analyses were conducted to evaluate robustness of findings: excluding RCTs at overall high risk of bias (RoB 2 “high risk” judgement); using a fixed-effect (inverse-variance) model; using ORs instead of RRs; restricting analyses to studies with a meaningful achieved between-group driving-pressure separation (absolute ΔP difference ≥3 cmH₂O); and excluding trials in which the ΔP target was achieved predominantly via protocolised PEEP titration (PEEP-driven strategy, as classified by the stated intervention protocol), with analyses repeated in trials using primarily tidal-volume reduction (Vt-driven strategy). To assess the impact of eligibility scope, we conducted a prespecified exploratory sensitivity analysis broadening the inclusion criteria to additionally incorporate RCTs of individualised PEEP titration strategies that achieved a demonstrably lower driving pressure in the intervention arm, even when DP minimisation was not explicitly stated as the principal decision rule. This broadened analysis was intended to explore whether directional findings were consistent across a less strictly defined intervention construct, but it does not represent confirmatory evidence for driving pressure-guided ventilation, because the additional trials evaluate mechanistically different strategies in which lower driving pressure is a downstream physiological consequence rather than the explicit target. Results from this broadened analysis were therefore reported and interpreted separately from the primary analysis, as exploratory supportive evidence only. Where subgroup and sensitivity analyses were underpowered due to limited study numbers, findings were reported descriptively and interpreted with appropriate caution.

## Results

### Study selection

A total of 282 records were identified through database and registry searches, including 269 from databases (25 from PubMed, 75 from Embase, 71 from CENTRAL, 46 from Web of Science Core Collection, and 52 from Scopus) and 13 from registers (6 from ClinicalTrials.gov and 7 from the WHO International Clinical Trials Registry Platform). After removal of 122 duplicates, 160 unique records remained for title and abstract screening. Of these, 118 records were excluded at the title/abstract stage, and 42 full-text articles were assessed for eligibility.

After full-text review, 32 articles were excluded. The most common reasons for exclusion were absence of an eligible comparator (*n* = 12), failure to meet the predefined comparison framework (*n* = 8; these studies were retained for narrative discussion only), study context outside one-lung ventilation for thoracic surgery (*n* = 6), registry entries without corresponding peer-reviewed full-text publications (*n* = 4), and absence of extractable outcome data (*n* = 2).

**Figure 1:**
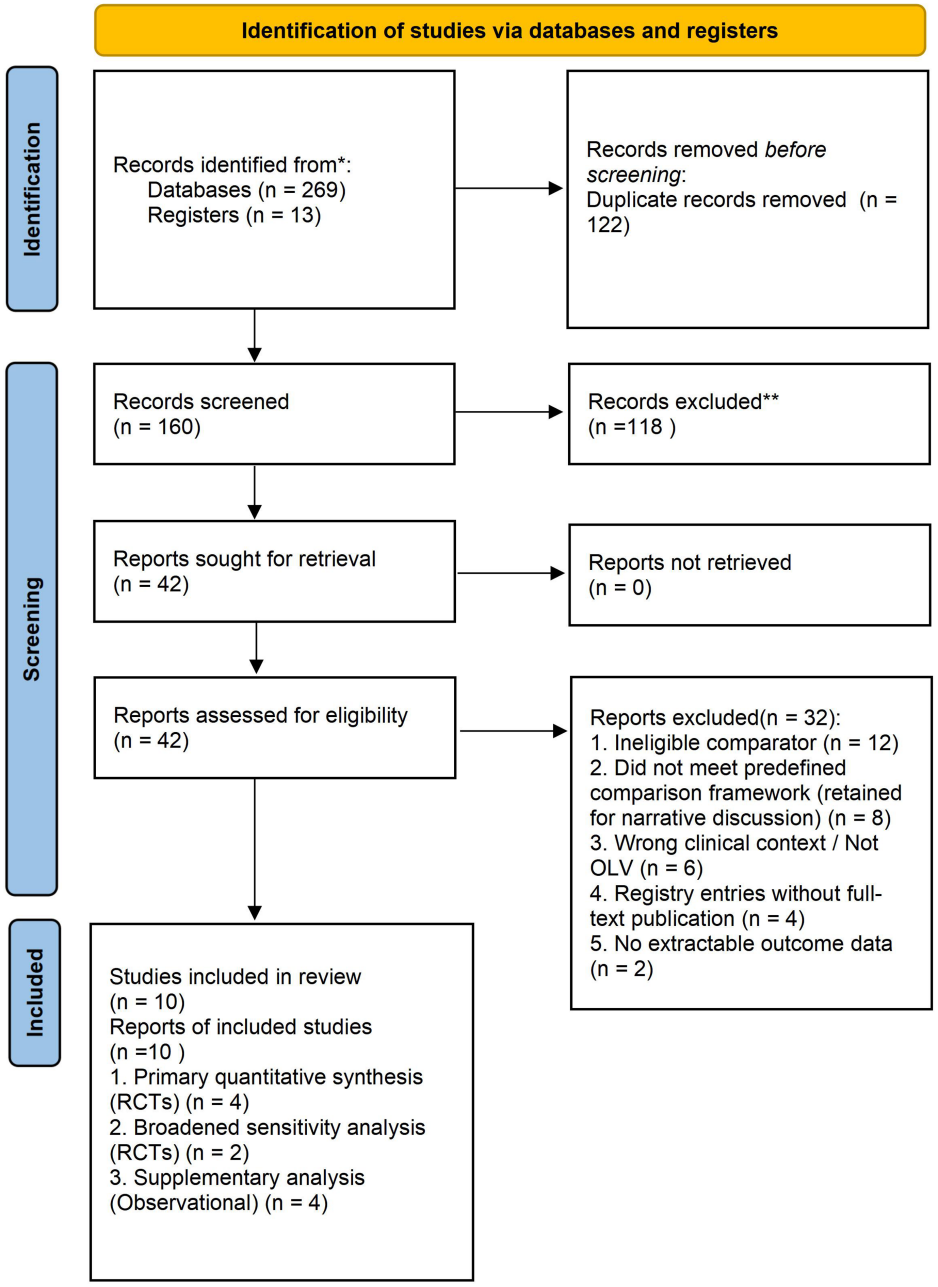
PRISMA 2020 flow diagram of study identification, screening, and inclusion. A total of 282 records were identified from database and registry searches, of which 160 unique records remained after de-duplication. After title/abstract screening and full-text review, four randomized controlled trials were included in the primary quantitative synthesis, two additional randomized trials were retained for the exploratory broadened sensitivity analysis, four observational studies were included as supplementary evidence, and eight further randomized trials were retained for narrative discussion only.

Ultimately, four randomized controlled trials met the prespecified criteria for inclusion in the primary quantitative synthesis comparing explicit driving pressure-guided ventilation with non-driving-pressure-guided ventilation during one-lung ventilation (16–19). In addition, two randomized trials evaluating individualised ventilation strategies that were not explicitly driving pressure-targeted—but in which lower driving pressure was observed as a downstream physiological consequence—were retained for the exploratory broadened sensitivity analysis (20, 21). Four observational studies were included as supplementary non-randomized evidence and were synthesized separately without pooled effect estimation (22–25). Eight further randomized trials evaluating broader protective ventilation strategies, individualised PEEP approaches, or alternative ventilatory modes were identified as contextually relevant but did not meet the predefined comparison framework for quantitative synthesis; these studies were retained for narrative discussion only (26–33). The study selection process is summarized in Figure 1.

### Characteristics of included studies

#### Primary randomized trials

The four randomized controlled trials included in the primary quantitative synthesis (16–19) were published between 2019 and 2026 and enrolled a total of 654 analysed patients, with sample sizes ranging from 59 to 292 patients per trial. Three trials were conducted in China and one in South Korea, and all four were single-centre studies. In terms of surgical population, two trials focused on lung resection, one enrolled patients undergoing minimally invasive oesophagectomy, and one included a broader thoracic surgical population comprising both lung and oesophageal procedures.

All four trials explicitly used driving pressure as the principal ventilatory target during one-lung ventilation. In three trials (16–18), individualized positive end-expiratory pressure was titrated to achieve the lowest driving pressure, typically following a recruitment manoeuvre or stepwise PEEP trial. In the remaining trial (19), tidal volume was titrated to maintain driving pressure within a prespecified target range of 8–10 cmH₂O. Across the four studies, the intervention strategy was therefore consistently based on either driving pressure minimisation or maintenance within a defined low range. By contrast, comparator groups received conventional lung-protective ventilation without driving pressure-guided adjustment, generally using fixed positive end-expiratory pressure of 4–5 cmH₂O and standard tidal-volume settings.

The primary clinical outcome of interest across trials was postoperative pulmonary complications, which were reported in all four studies using study-defined composite or clinically relevant pulmonary endpoints. All four trials also reported intraoperative respiratory mechanics, including driving pressure, plateau pressure, and dynamic or respiratory-system compliance, together with oxygenation indices during one-lung ventilation. Additional reported outcomes varied across studies and included ICU and hospital length of stay, individual pulmonary complications, and, in one trial, bronchoalveolar lavage fluid interleukin-6 as a biomarker of lung injury. The achieved between-group separation in driving pressure during one-lung ventilation was approximately 1–3 cmH₂O across the included trials, indicating that the intervention produced a measurable physiological contrast in all studies. Detailed characteristics of the primary randomized trials are presented in Table 1.

**Table 1:** Characteristics of included randomized controlled trials.

Study	Country	Design	Analysed sample, *n* (Int/Ctrl)	Surgical population	Intervention strategy	DP target/rule	Comparator	OLV duration (Int/Ctrl)	Achieved DP (Int/Ctrl)	PPC definition/window	PPCs (Int vs. Ctrl)	Other reported outcomes
Primary quantitative synthesis (strict DP-guided)												
Park (16)	South Korea	Single-centre RCT	292 (147/145)	Mixed thoracic surgery (lung resection + oesophagectomy)	DP-guided PEEP titration after recruitment manoeuvre	Lowest DP (no fixed threshold)	Fixed PEEP 5 cmH₂O; VT 6 mL/kg PBW	105 [79–137]/104 [84–140] min	9 [8–10]/10 [9–11] cmH₂O	MGS ≥ 4; 3-day	5.5% vs. 12.2% (*p* = 0.047)	Respiratory mechanics, PaO₂, ICU LOS, hospital LOS
Yu (17)	China	Single-centre RCT	207 (104/103)	Lung resection (VATS or thoracotomy)	DP-guided PEEP titration after recruitment manoeuvre	Lowest DP (no fixed threshold)	Fixed PEEP 4 cmH₂O; VT 6 mL/kg PBW	115.7 ± 49.8/125.3 ±120.5 min	10 [8–11]/13 [10–15] cmH₂O	MGS ≥ 4; 3-day and 7-day	4% vs. 13% at 3 days (*p* = 0.021); no significant difference at 7 days	Respiratory mechanics, PaO₂, Cdyn, ICU LOS, hospital LOS
Zhang (18)	China	Single-centre RCT	59 (30/29)	Minimally invasive oesophagectomy	DP-guided PEEP titration after manual recruitment manoeuvre	Lowest DP (no fixed threshold)	Fixed PEEP 5 cmH₂O; VT 6–8 mL/kg PBW	137.9 ± 27.3/142.2 ±36.6 min	10.3 ± 1.5/12.8 ± 1.9 cmH₂O	ECCG-defined PPCs; 7-day	24.1% vs. 43.3% (P = 0.12)	Respiratory mechanics, PaO₂, Cdyn, hospital LOS
Yan (19)	China	Single-centre RCT	96 (46/50)	Lung resection (lobectomy/segmentectomy/wedge resection)	DP-guided VT titration	DP 8–10 cmH₂O	Fixed VT 8 mL/kg PBW; fixed PEEP 8 cmH₂O	90 [72.5–100]/90 [70–100] min	9.2 ± 1.3/11.6 ± 1.8 cmH₂O	Composite PPCs; 7-day	39.1% vs. 38.0%; RR 0.98 (95% CI 0.66–1.45), *p* = 0.911	BALF IL-6, respiratory mechanics, PaO₂/FiO₂, mechanical power, hospital LOS
Broadened sensitivity analysis (individualised PEEP; DP reduction as consequence rather than target)												
Zhang (20)	China	Single-centre RCT	58 (29/29)	VATS lobectomy	Individualised PEEP titration to maximum pulmonary compliance	Not DP-targeted	Fixed PEEP 5 cmH₂O	104 ± 13/102 ± 16 min	11.7 ± 3.3/14.8 ± 2.4 cmH₂O	Composite PPCs (pulmonary infection, atelectasis, hypoxemia); 3-day	6/29 vs. 11/29 (*p* > 0.05)	Respiratory mechanics, oxygenation, compliance
Wang (21)	China	Single-centre RCT	392 (195/197)	Lung cancer surgery (lobectomy/segmentectomy/wedge resection); older adults	Individualised PEEP guided by EIT	Not DP-targeted	Fixed PEEP 5 cmH₂O	NR	12 [11–14]/15 [13–18] cmH₂O	Composite PPCs within 7 days (pneumonia, atelectasis, hypoxemia/respiratory failure, pleural effusion, pneumothorax)	28% vs. 25% (*p* = 0.60)	Respiratory mechanics, oxygenation, EIT-derived regional ventilation

BALF, bronchoalveolar lavage fluid; Cdyn, dynamic compliance; CI, confidence interval; Ctrl, control; DP, driving pressure; ECCG, Esophagectomy Complications Consensus Group; EIT, electrical impedance tomography; Int, intervention; LOS, length of stay; MGS, Melbourne Group Scale; NR, not reported; OLV, one-lung ventilation; PaO₂, arterial oxygen tension; PBW, predicted body weight; PEEP, positive end-expiratory pressure; PPCs, postoperative pulmonary complications; RCT, randomized controlled trial; RR, risk ratio; VATS, video-assisted thoracoscopic surgery; VT, tidal volume. Data are presented as mean ± standard deviation, median [interquartile range], or n/N (%) as reported by the original investigators. All sample sizes refer to the analysed population. The upper panel includes the four trials meeting the strict eligibility criteria for the primary quantitative synthesis (explicit driving pressure-guided ventilation). The lower panel includes two additional trials retained for a prespecified broadened sensitivity analysis, in which individualised PEEP strategies achieved lower driving pressure as a physiological consequence rather than as the prespecified decision target.

#### Additional trials included in the broadened sensitivity analysis

Two additional randomized trials (20, 21) were retained for the exploratory broadened sensitivity analysis. Together, these studies contributed 450 analysed patients, with sample sizes ranging from 58 to 392 patients. Both trials were conducted in China, both were single-centre studies, and both enrolled patients undergoing thoracic surgery requiring one-lung ventilation.

In both studies, the intervention consisted of individualized positive end-expiratory pressure titration, whereas the comparator group received fixed positive end-expiratory pressure of 5 cmH₂O. However, unlike the four primary randomized trials, neither study explicitly prespecified driving pressure minimisation as the decision rule for ventilator adjustment. In Wang et al. (21), PEEP titration was guided by electrical impedance tomography to balance regional overdistension and recruitable collapse, whereas in Zhang et al. (20), PEEP was individualized according to maximum pulmonary compliance. In both trials, lower driving pressure was observed in the intervention group, but this reduction represented a physiological consequence of individualized ventilation rather than the formal intervention target. For this reason, these studies were not included in the strict primary analysis but were considered sufficiently relevant for an exploratory broadened sensitivity analysis examining whether inclusion of driving-pressure-lowering strategies yielded directionally consistent findings. Their characteristics are also summarized in Table 1.

### Supplementary observational studies

Four observational cohort studies were included as supplementary non-randomized evidence (22–25). Together, these studies enrolled 4,339 patients, with sample sizes ranging from 197 to 3,386 in the driving-pressure-related analytic cohorts. Two studies were prospective (22, 23) and two were retrospective (24, 25). The study populations included patients undergoing lung resection, oesophagectomy, or mixed thoracic surgery under one-lung ventilation.

These studies differed conceptually from the randomized trials because they examined intraoperative driving pressure as an exposure variable rather than comparing a driving pressure-guided ventilation strategy with a conventional control strategy. Driving pressure was analysed as a continuous or time-weighted variable in all four studies, although the exact exposure definition varied, including time-weighted average values during one-lung ventilation, median driving pressure during one-lung ventilation, or intraoperative mean values across different ventilation phases. Postoperative pulmonary complications were the main outcome of interest across all studies, but definitions and follow-up windows varied modestly between cohorts.

Adjusted effect estimates were reported in three studies. Cirenei et al. (25) found that higher driving pressure during one-lung ventilation was independently associated with postoperative pulmonary complications after oesophagectomy. Yang et al. (24) likewise reported an independent association between higher driving pressure and postoperative pulmonary complications in a large thoracic surgical cohort. By contrast, Okahara et al. (22) did not identify driving pressure as an independent predictor in adjusted analyses, and Uhlig et al. (23) did not demonstrate a clear association between postoperative pulmonary complications and intraoperative mechanical ventilation settings in the thoracic subgroup. Because of this exposure–outcome design and heterogeneity in covariate adjustment, exposure definition, and outcome ascertainment, these studies were synthesized narratively rather than pooled quantitatively. Their characteristics are presented in Table 2.

**Table 2:** Characteristics of supplementary observational studies.

Study	Country	Design	Centre	Analysed sample, *n*	Surgical population	DP exposure definition	PPC definition/window	Adjusted effect estimate	DP independently associated with PPCs?	Main adjustment covariates
Cirenei (25)	France	Retrospective cohort	Single-centre	454	Oesophagectomy	Continuous; time-weighted average DP during OLV and TLV	Composite PPCs; 7-day	OR 1.17 per SD increase during OLV, 95% CI 1.05–1.30, P=0.005	Yes	Age, history of neoadjuvant therapy, Charlson Comorbidity Index, duration of surgery, intraoperative lactate
Yang (24)	South Korea	Retrospective cohort	Single-centre	3386	VATS thoracic surgery, predominantly lung resection	Continuous; time-weighted median DP during OLV	Composite PPCs; 7-day	OR 1.047 per 1 cmH₂O increase, 95% CI 1.019–1.075, P=0.001	Yes	Age, ASA class, smoking history, duration of surgery, duration of OLV, intraoperative blood loss
Okahara (22)	Japan	Prospective cohort	Multicentre (2 centres)	197	Thoracic surgery, predominantly lung resection	Continuous; time-weighted average ΔP during the first 2 h of OLV	Composite PPCs; 7-day	OR 1.03 per 1 cmH₂O increase, 95% CI 0.91–1.16, P=0.64	No	Age, sex, BMI, preoperative pulmonary function/FEV1, duration of surgery, fluid administration
Uhlig (22)	Multinational (29 countries)	Prospective cohort	Multicentre	302	Mixed thoracic surgery, mainly lung and pleural procedures	Continuous; intraoperative mean DP across ventilation phases	Composite PPCs; 5-day	No adjusted DP effect estimate reported for the thoracic subgroup	No clear association	ARISCAT risk score, type of surgery, tidal volume, PEEP

ASA, American Society of Anesthesiologists; ARISCAT, Assess Respiratory Risk in Surgical Patients in Catalonia; BMI, body mass index; CI, confidence interval; DP, driving pressure; FEV₁, forced expiratory volume in one second; OLV, one-lung ventilation; OR, odds ratio; PPCs, postoperative pulmonary complications; SD, standard deviation; TLV, two-lung ventilation; VATS, video-assisted thoracoscopic surgery. These four observational cohort studies examined intraoperative driving pressure as an exposure variable rather than comparing a driving pressure-guided strategy with a conventional control; accordingly, they were synthesised narratively and not pooled quantitatively with the randomized trials.

### Risk of bias assessment

#### Randomized controlled trials

**Figure 2:**
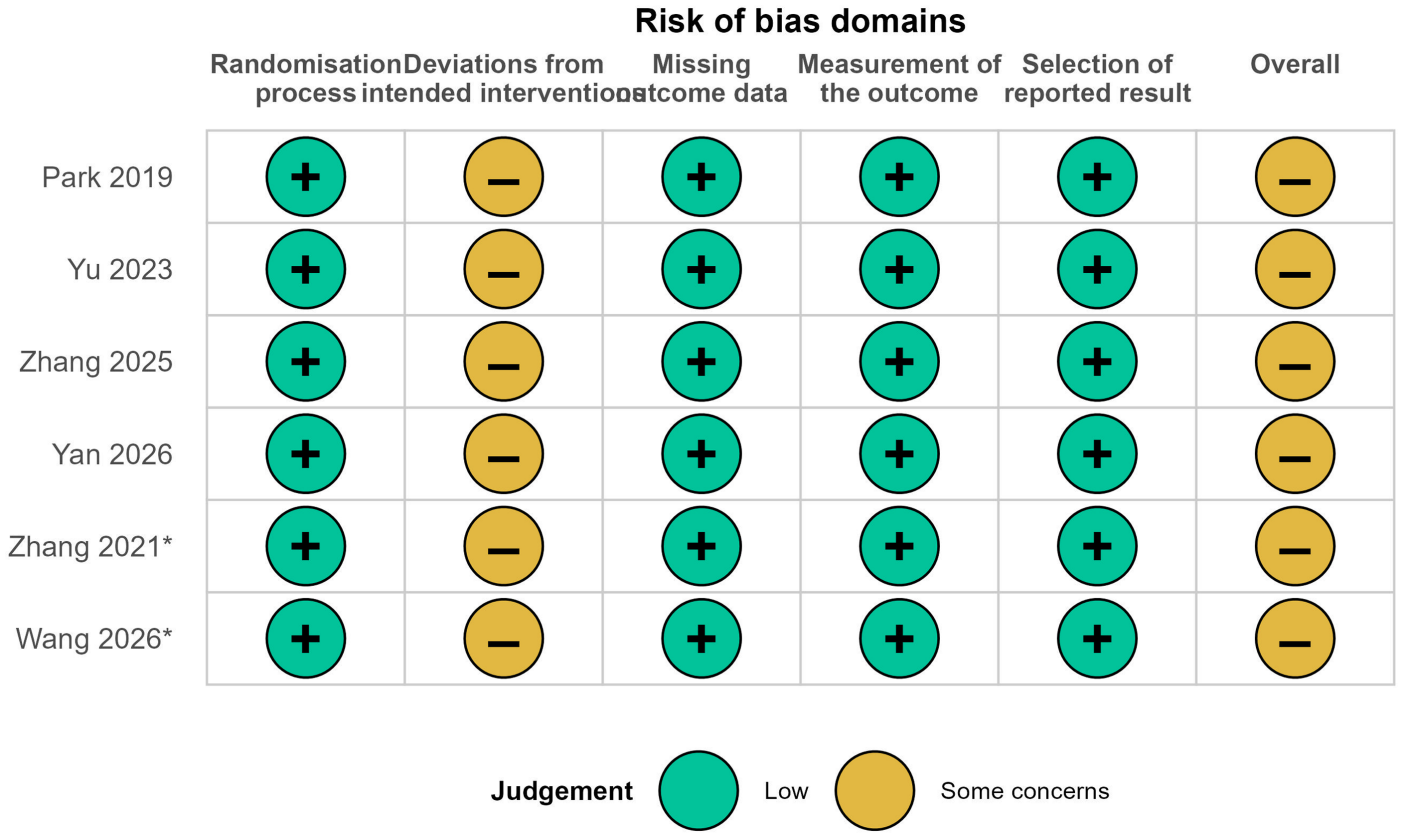
Risk of bias assessment for included randomized controlled trials using the Cochrane Risk of Bias 2 (RoB 2) tool. The four primary trials (16–19) and two additional trials retained for the exploratory broadened sensitivity analysis [(20, 21); marked with asterisks] are shown. Each domain was judged as low risk of bias (+, green) or some concerns (−, yellow). All six trials were judged as having some concerns overall, driven primarily by the practical impossibility of blinding the treating anaesthesiologist to intraoperative ventilator management (Domain 2: deviations from intended interventions).

Risk-of-bias assessments for the randomized trials are summarized in Figure 2 and detailed in Supplementary Table S2. Among the four trials included in the primary quantitative synthesis (16–19), all were judged to have some concerns overall according to the RoB 2 tool (12). Across these studies, the randomization process was generally considered to be at low risk of bias, as adequate sequence generation and allocation concealment were described and baseline characteristics were balanced between groups. Bias due to missing outcome data was also judged to be low, because follow-up was highly complete and attrition was minimal. Outcome measurement was considered to be at low risk in all trials, as postoperative pulmonary complications were assessed using prespecified clinical criteria and postoperative assessors were blinded to group allocation. Reporting bias was likewise judged to be low because all trials had trial registration and reported outcomes that were consistent with the prespecified protocols. The overall judgement of some concerns was driven primarily by bias due to deviations from intended interventions, reflecting the practical impossibility of blinding the treating anaesthesiologist to intraoperative ventilator management.

The two additional trials retained for the exploratory broadened sensitivity analysis showed broadly comparable risk-of-bias profiles (20, 21), with the overall judgement of some concerns likewise driven by the practical impossibility of blinding intraoperative ventilator management.

#### Observational studies

Risk-of-bias assessments for the four supplementary observational studies are detailed in Supplementary Table S3 (ROBINS-I, pre-specified) and Supplementary Table S4 (QUIPS, post-hoc sensitivity appraisal). According to the pre-specified ROBINS-I framework (13), three studies were judged to be at moderate risk of bias overall and one study at serious risk of bias. The principal source of bias across these studies was confounding, because intraoperative driving pressure was analysed as an exposure variable within routine clinical practice rather than as an allocated intervention.

Among the four studies, Yang et al. (24), Cirenei et al. (25), and Okahara et al. (22) were judged to be at moderate risk of bias overall. In these cohorts, the main concerns related to residual confounding, particularly incomplete adjustment for potentially important factors such as body mass index, baseline pulmonary function, or other perioperative variables. Additional concerns arose from the observational design itself, including participant selection and the retrospective ascertainment of exposure or outcome data in some studies. By contrast, Uhlig et al. (23) was judged to be at serious risk of bias overall because the thoracic subgroup analysis did not adjust for key covariates, resulting in a serious risk of confounding bias. Outcome measurement was generally less problematic across the observational studies because postoperative pulmonary complications were defined using clinically relevant postoperative endpoints, although exposure definitions and adjustment strategies varied across cohorts. A post-hoc sensitivity appraisal using the QUIPS tool (Supplementary Table S4) yielded broadly concordant overall judgements—three studies rated as moderate risk and one (23) rated as high risk—with study confounding being the predominant concern across both frameworks, supporting the robustness of the narrative synthesis conclusions.

### Quantitative synthesis of randomized trials

#### Primary analysis

The prespecified co-primary outcome of clinically significant intraoperative haemodynamic adverse events was not consistently reported as a defined adverse event across the included trials. As documented in the Methods section, this represents a deviation from the PROSPERO-registered protocol, identified during data extraction and documented before quantitative synthesis began. Although five of the six randomized trials reported intraoperative vasopressor use, these data were recorded as descriptive intraoperative characteristics rather than as prespecified safety endpoints. This outcome was therefore reclassified as an exploratory secondary outcome (intraoperative vasopressor use) and is reported separately. Only postoperative pulmonary complications, therefore, could be assessed as originally registered in the PROSPERO co-primary framework.

##### Postoperative pulmonary complications

**Figure 3:**
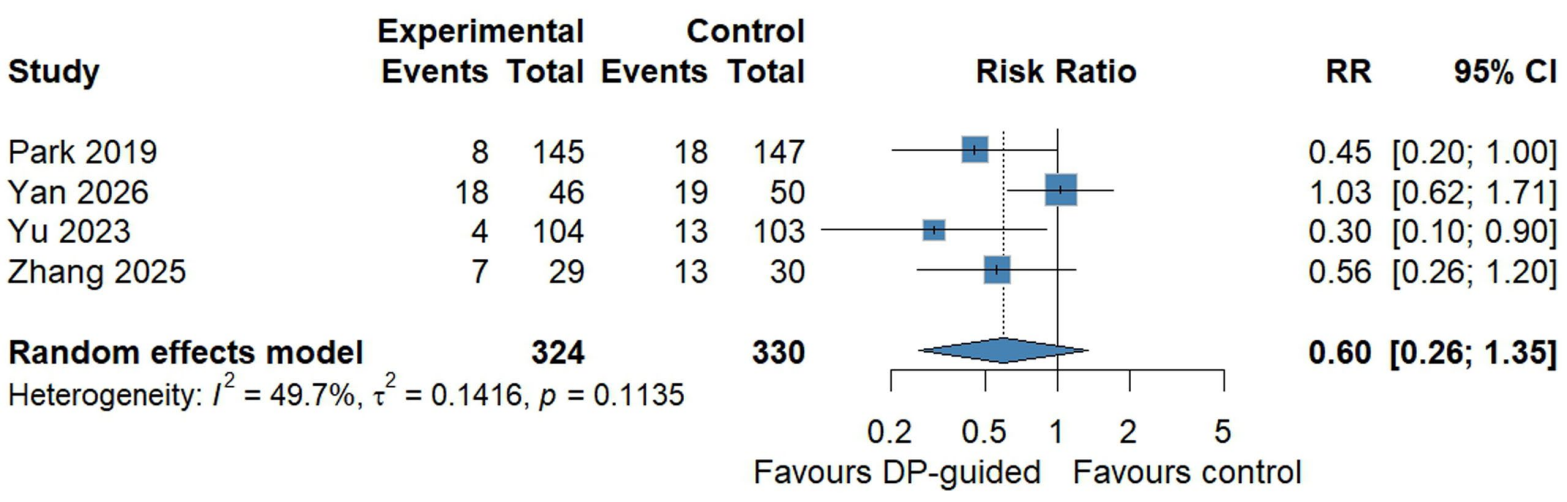
Forest plot of the primary meta-analysis for postoperative pulmonary complications. Four randomized controlled trials comparing driving pressure-guided ventilation with conventional ventilation during one-lung ventilation were pooled using a random-effects model with restricted maximum likelihood estimation and Hartung-Knapp adjustment. The pooled risk ratio was 0.60 (95% CI: 0.26–1.35; *p* = 0.14), with moderate statistical heterogeneity (*I*^2^ = 49.7%; Cochran’s *Q* = 5.96, *p* = 0.11). The diamond represents the pooled effect estimate; horizontal lines represent 95% confidence intervals for individual studies. The dashed vertical line indicates the null effect (RR = 1.0).

Four randomized controlled trials (654 patients; 324 in driving pressure-guided groups and 330 in control groups) contributed to the primary meta-analysis of postoperative pulmonary complications (16–19). The pooled effect did not reach statistical significance in the primary random-effects meta-analysis with Hartung-Knapp adjustment (RR: 0.60; 95% CI: 0.26–1.35; *p* = 0.14; Figure 3). The absolute PPC incidence ranged from 3.8 to 39.1% in the intervention groups and from 12.4 to 43.3% in the control groups. This variation reflected substantial differences in PPC definitions and ascertainment windows across trials: Park et al. (16) and Yu et al. (17) used a Melbourne Group Scale ≥4 threshold assessed within the first 3 postoperative days; Zhang et al. (18) used an ECCG-aligned composite assessed within 7 days; and Yan et al. (19) used a study-specific composite endpoint. This heterogeneity in both the operational PPC definition and the ascertainment window likely contributes to between-study variability independent of the intervention effect. Statistical heterogeneity was moderate (*I*^2^ = 49.7%; Cochran’s *Q* = 5.96, *p* = 0.11).

Visual inspection of the forest plot indicated that the effect was directionally consistent across three of the four trials (16–18), with individual risk ratios ranging from 0.30 to 0.56. By contrast, Yan et al. (19) showed no apparent effect (RR 1.03, 95% CI 0.62–1.71) and was the main contributor to between-study heterogeneity. Notably, Yan et al. (19) was the only trial to use a tidal-volume-titration strategy to maintain driving pressure within 8–10 cmH₂O rather than a PEEP-titration approach, and PPC rates were high in both groups (39.1 and 38.0%). However, with only four trials and a single Vt-titration study, this apparent pattern is purely descriptive: formal quantitative interaction testing was not feasible, and the pattern could reflect confounding by trial-level differences other than the implementation pathway (e.g., different patient populations, baseline PPC risk, or PPC definitions). This observation is therefore presented as hypothesis-generating only.

The pooled absolute risk difference for PPCs was −7.6% (95% CI − 13.5 to −1.6%; *p* = 0.027), corresponding to a number needed to treat of 13, although this metric should be interpreted with caution given the substantial variation in baseline PPC risk across trials (12.4 to 43.3% in the control arms). Heterogeneity was absent on the absolute scale (*I*^2^ = 0%; *Q* = 1.86, *p* = 0.60), in contrast to the moderate heterogeneity observed for the relative effect estimate. The absolute and relative effect scales therefore yielded discrepant statistical conclusions—the RR estimate was imprecise and crossed the null, whereas the RD was statistically significant. This discrepancy reflects the limited number of trials, heterogeneous baseline event rates, and differential susceptibility of the two scales to such heterogeneity, rather than convergent support for an intervention effect.

### Broadened sensitivity analysis

As an exploratory extension of the primary analysis, the two additional trials of individualised PEEP strategies with documented lower driving pressure (20, 21) were included alongside the four primary trials. In these two trials, lower driving pressure was observed as a physiological consequence of individualised ventilation rather than as the explicit intervention target, and their inclusion therefore broadens the intervention construct beyond the strict primary eligibility framework. The pooled estimate from six randomized trials (1,104 patients) remained directionally consistent but did not reach statistical significance (RR: 0.70; 95% CI: 0.42–1.15; *p* = 0.12; *I*^2^ = 53.7%; Supplementary Figure S1). The slight attenuation of the point estimate compared with the primary analysis was driven mainly by Wang et al. (21) (RR: 1.09; 95% CI: 0.78–1.52), in which PPC rates were similar between the individualised and fixed PEEP groups. This result should be interpreted as directionally supportive rather than as independent confirmation of effect, because the intervention definition differs from that of the primary analysis.

### Additional robustness checks

A prespecified sensitivity analysis using a fixed-effect model yielded a statistically significant pooled estimate for PPCs (RR: 0.61; 95% CI: 0.43–0.87; *p* = 0.007), whereas the primary Hartung-Knapp random-effects analysis did not. When odds ratios were used as an alternative effect measure, the direction and magnitude of the effect were consistent (OR: 0.51; 95% CI: 0.20–1.29; *p* = 0.10; *I*^2^ = 30.6%).

Of the prespecified subgroup and sensitivity analyses, only two sensitivity analyses (fixed-effect model and OR as alternative effect measure) and one exploratory extension (broadened eligibility) were quantitatively estimable. The remaining prespecified subgroup analyses and sensitivity analyses—including restriction to trials with a between-group driving pressure separation of ≥3 cmH₂O, exclusion of predominantly PEEP-driven strategies, exclusion of high-risk-of-bias trials, and all six prespecified subgroup comparisons—could not be conducted as formal quantitative analyses owing to the limited number of included trials (k = 4) and, in one case, the absence of any trial meeting the exclusion criterion. The full mapping of planned versus actually estimable analyses is presented in the Supplementary material. Planned analyses that were not quantitatively feasible are reported descriptively in Supplementary Table S6.

### Secondary outcomes in randomized trials

#### Oxygenation

Intraoperative oxygenation was reported in all four primary trials (15–18), although measurement timepoints and indices varied across studies, precluding quantitative pooling. Park et al. (16) reported PaO₂ at 15 min of one-lung ventilation (249.8 ± 105.1 vs. 224.2 ± 102.7 mmHg; intervention vs. control), Yu et al. (17) reported PaO₂ at 30 min (184.1 ± 89.6 vs. 158.9 ± 82.6 mmHg), and Zhang et al. (18) reported PaO₂ at both 30 min (85.0 ± 16.0 vs. 76.6 ± 14.9 mmHg) and 60 min (83.4 ± 16.5 vs. 75.1 ± 12.1 mmHg) of one-lung ventilation. Yan et al. (19) reported PaO₂/FiO₂ ratio at 45 min (median 200.50 [IQR 119.25–303.25] vs. 181.00 [141.00–238.00] mmHg). Across the four trials, oxygenation tended to be numerically higher in the driving pressure-guided groups, although reporting metrics, measurement timepoints, and statistical presentation varied across studies. Refractory hypoxaemia was explicitly reported only by Zhang et al. (20), in which no events occurred in either group.

### Ventilatory mechanics and intervention fidelity

All four primary trials reported achieved driving pressure during one-lung ventilation, confirming that the intervention produced a measurable physiological separation between groups (16–19). The between-group difference in driving pressure ranged from approximately 1 cmH₂O [Park et al. (16): median 9 (8–10) vs. 10 (9–11) cmH₂O] to approximately 2.5 cmH₂O [Zhang et al. (18): 10.3 ± 1.5 vs. 12.8 ± 1.9 cmH₂O; Yan et al. (19): 9.2 ± 1.3 vs. 11.6 ± 1.8 cmH₂O], with Yu et al. (17) reporting a 3 cmH₂O separation [median 10 (8–11) vs. 13 (10–15) cmH₂O]. All four trials demonstrated lower achieved driving pressure in the intervention group, with clear between-group physiological separation during one-lung ventilation.

Plateau pressure was reported by two trials. In Park et al. (16), Pplat was lower in the driving pressure-guided group [median 12 (11–14) vs. 15 (14–16) cmH₂O], whereas Yu et al. (17) reported similar Pplat between groups [median 16 (15–18) vs. 17 (14–19) cmH₂O]. PEEP levels in the intervention groups varied according to the individualisation protocol: the titrated PEEP was lower than the fixed comparator in Park et al. (16): [median 3 (2–5) vs. 5 cmH₂O] but higher in Yu et al. (17): [median 7 (6–8) vs. 4 cmH₂O]. In Yan et al. (19), PEEP was fixed at 8 cmH₂O in both groups because the intervention targeted driving pressure via tidal-volume reduction rather than PEEP titration.

Dynamic compliance was reported by two trials and was consistently higher in the driving pressure-guided groups [Yu et al. (17): 38.2 ± 10.0 vs. 30.2 ± 9.2 mL/cmH₂O; Zhang et al. (18): approximately 36 vs. 27 mL/cmH₂O, as reported graphically without formal dispersion measures].

### Other clinical and resource use outcomes

Hospital length of stay was reported by all four primary trials and showed no clear between-group difference in any study [Park et al. (16): median 6 (5–9) vs. 6 (5–9) days; Yu et al. (17): 11.8 ± 3.9 vs. 12.1 ± 4.6 days; Zhang et al. (18): median 16.5 vs. 14.5 days; Yan et al. (19): median 7.50 (7.00–9.00) vs. 8.00 (6.00–9.00) days]. ICU length of stay was reported by two trials, with no significant differences [Park et al. (16): median 22 (18–25) vs. 21 (18–25) h; Yu et al. (17): 19.1 ± 10.5 vs. 20.3 ± 12.7 h]. Mortality was reported only by Park et al. (16): (1/145 vs. 2/147) and was too rare to draw any conclusions.

Intraoperative vasopressor use was reported descriptively in five of the six randomized trials but could not be pooled because of substantial heterogeneity in outcome definition. Park et al. (16) and Yu et al. (17) reported the number of patients receiving ephedrine for hypotensive episodes (Park: 18/145 vs. 23/147; Yu: 10/104 vs. 14/103), whereas Zhang et al. (18) and Yan et al. (19) reported overall intraoperative vasopressor use including both prophylactic and rescue administration [Zhang et al. (18): 27/29 vs. 21/30; Yan et al. (19): 37/46 vs. 35/50]. The resulting event rates differed markedly across studies (approximately 10–16% vs. 80–93%), indicating substantial definitional heterogeneity and precluding meaningful quantitative synthesis. Within each definition category, the direction of effect was numerically consistent, with slightly lower vasopressor use in the driving pressure-guided groups, but no individual comparison reached statistical significance.

One trial (19) reported bronchoalveolar lavage fluid interleukin-6 concentration as a biomarker of lung injury. IL-6 levels were significantly lower in the driving pressure-guided group (median 5.31 [3.62] vs. 7.37 [5.21] pg./mL; between-group difference −0.46, 95% CI − 0.86 to −0.05; *p* = 0.025), consistent with a lower intraoperative inflammatory signal in the intervention group.

### Supplementary evidence, subgroup analyses, sensitivity analyses, and publication bias

#### Observational cohort evidence

Four observational studies (22–25) examined the association between intraoperative driving pressure and postoperative pulmonary complications in thoracic surgical patients undergoing one-lung ventilation. Because these studies analysed driving pressure as a continuous exposure variable rather than comparing a driving pressure-guided strategy with a conventional control, they were synthesised narratively.

Two observational cohorts identified driving pressure as an independent predictor of postoperative pulmonary complications. Cirenei (25) (*n* = 454, oesophagectomy) reported an adjusted odds ratio of 1.17 per standard deviation increase in time-weighted average driving pressure during one-lung ventilation (95% CI 1.05–1.30; *p* = 0.005). Yang (24) (*n* = 3,386, predominantly VATS lung resection) reported an adjusted odds ratio of 1.047 per 1 cmH₂O increase in time-weighted median driving pressure during one-lung ventilation (95% CI: 1.019–1.075; *p* = 0.001). By contrast, Okahara (22) (*n* = 197, prospective multicentre cohort) did not identify driving pressure as an independent predictor in adjusted analyses (OR 1.03 per 1 cmH₂O, 95% CI 0.91 to 1.16; *p* = 0.64), and Uhlig (23) (*n* = 302, LAS VEGAS thoracic subgroup) did not demonstrate a clear association between postoperative pulmonary complications and intraoperative ventilation settings in the thoracic subgroup; this subgroup analysis was not adjusted for covariates.

Overall, the observational evidence was mixed: two studies supported an independent association between higher driving pressure and increased PPC risk, whereas two did not confirm this association. The direction of the positive observational findings was broadly consistent with the point estimate from the primary randomized analysis.

#### Subgroup analyses

Prespecified subgroup analyses were planned to explore potential effect modifiers, including driving pressure-guided strategy type (tidal-volume-driven vs. PEEP-driven), driving pressure target threshold, recruitment manoeuvre use, surgical category, background tidal volume strategy, and high-risk lung populations. However, with only four trials in the primary analysis, none of these subgroup comparisons could be conducted as formal quantitative interaction tests. Descriptively, three of the four primary trials used a PEEP-titration approach to minimise driving pressure (16–19), and all three showed point estimates favouring the intervention (RR range: 0.30–0.56). The single trial using a tidal-volume-titration approach (19) showed no apparent effect (RR: 1.03). Formal quantitative interaction testing was not feasible with only four trials, and this pattern is presented descriptively and as hypothesis-generating only, not as evidence of differential efficacy between implementation pathways.

#### Sensitivity analyses

The results of prespecified sensitivity analyses are summarised together with the primary findings. The fixed-effect model yielded a statistically significant result (RR: 0.61; 95% CI: 0.43–0.87; *p* = 0.007), whereas the primary random-effects model with Hartung–Knapp adjustment did not (RR: 0.60; 95% CI: 0.26–1.35; *p* = 0.14). Using odds ratios as an alternative effect measure produced consistent results (OR: 0.51; 95% CI: 0.20–1.29; *p* = 0.10). The absolute risk difference was statistically significant (RD: −7.6, 95% CI − 13.5 to −1.6%; *p* = 0.027; NNT = 13), although this metric should be interpreted with caution given the substantial variation in baseline PPC risk across trials. The exploratory broadened sensitivity analysis incorporating two additional trials of individualised PEEP strategies (six trials, 1,104 patients) yielded a directionally consistent but non-significant result (RR: 0.70, 95% CI: 0.42–1.15; *p* = 0.12), and is interpreted as exploratory supportive evidence rather than as confirmation of the primary result. Restriction to trials with a between-group driving pressure separation of ≥3 cmH₂O and exclusion of predominantly PEEP-driven strategies could not be formally tested because of the small number of studies and are reported descriptively in the Supplementary material. No trial was judged at overall high risk of bias, so the prespecified sensitivity analysis excluding high-risk-of-bias studies was not applicable.

#### Publication bias

Formal assessment of publication bias using funnel plots and Egger’s regression test was not performed because the number of studies in any single meta-analysis did not reach the recommended minimum of 10. Publication bias and small-study effects therefore cannot be excluded.

### Certainty of evidence

The certainty of evidence for postoperative pulmonary complications in the primary randomized analysis was assessed using the GRADE approach (15) and is summarised in Table 3. Evidence from four randomized controlled trials began at high certainty and was downgraded by two levels. The first downgrade was for inconsistency, owing to moderate statistical heterogeneity (*I*^2^ = 49.7%) and discordant effect direction in one trial (19). The second downgrade was for imprecision, as the pooled confidence interval for the relative effect was wide and crossed the null (RR: 0.60; 95% CI: 0.26–1.35), the total sample size was modest (654 patients, 100 events), and a clinically important benefit could be neither confirmed nor excluded. No downgrade was applied for risk of bias, because all included trials were judged as having some concerns rather than high risk of bias, with the main limitation relating to the impracticality of blinding ventilator management. No downgrade was applied for indirectness, as the study populations, interventions, comparators, and outcomes directly matched the prespecified PICO framework. Publication bias could not be formally assessed because only four trials were available, and no specific indicator prompted downgrading. The overall certainty of evidence for the effect of driving pressure-guided ventilation on postoperative pulmonary complications was therefore rated as low.

**Table 3:** Summary of findings and GRADE assessment for the co-primary outcome of postoperative pulmonary complications in the primary analysis of randomized controlled trials.

Outcome	Anticipated absolute effects (95% CI)	Relative effect (95% CI)	No. of participants (studies)	Certainty of the evidence (GRADE)	Interpretation
Postoperative pulmonary complications	76 fewer per 1000 (from 135 fewer to 16 fewer)*	RR 0.60 (0.26 to 1.35)	654 (4 RCTs) *Total events: 100*	⨁⨁◯◯ LOW	Driving pressure-guided ventilation during one-lung ventilation may reduce postoperative pulmonary complications, but confidence in this estimate is limited. The true effect may be substantially different from the observed estimate.

GRADE assessment details (reasons for downgrading)		
Domain	Judgement	Justification
Risk of bias	Not serious	All trials judged as 'some concerns' rather than 'high risk' of bias; the main limitation related to the inherent impracticality of blinding intraoperative ventilator management.
Inconsistency	Serious (-1)	Moderate statistical heterogeneity (*I*² = 49.7%) accompanied by a discordant effect direction in one trial (19).
Indirectness	Not serious	Populations, interventions, comparators, and outcomes directly matched the prespecified review PICO framework.
Imprecision	Serious (-1)	Wide confidence interval crossing the null effect line on the relative scale (RR 95% CI: 0.26–1.35). Total sample size (654 patients, 100 events) did not meet the optimal information size.
Publication bias	Undetected	Formal assessment via funnel plot was not possible due to the small number of studies (k = 4). No specific indicator prompted downgrading.

* The absolute effect was derived from the pooled risk difference analysis (RD −7.6%, 95% CI −13.5% to −1.6%; *p* = 0.027).

Certainty of evidence was assessed using the Grading of Recommendations Assessment, Development and Evaluation (GRADE) approach. Evidence from randomized controlled trials began at high certainty and was downgraded as appropriate. CI, confidence interval; GRADE, Grading of Recommendations Assessment, Development and Evaluation; NNT, number needed to treat; PICO, population–intervention–comparator–outcomes; RCT, randomized controlled trial; RD, risk difference; RoB 2, Cochrane Risk of Bias 2; RR, risk ratio.

The prespecified co-primary outcome of clinically significant intraoperative haemodynamic adverse events was not available for GRADE assessment because it was not consistently reported as a defined adverse event and was reclassified as an exploratory secondary outcome, as documented in the Methods section. GRADE assessments were therefore restricted to postoperative pulmonary complications in the primary randomized analysis and were not applied to secondary outcomes or supplementary observational evidence.

## Discussion

The primary random-effects meta-analysis of four randomized controlled trials did not demonstrate a statistically significant reduction in postoperative pulmonary complications with driving pressure-guided ventilation during one-lung ventilation (RR: 0.60; 95% CI: 0.26–1.35; *p* = 0.14), and the overall certainty of evidence was rated low according to GRADE (15), owing to inconsistency and imprecision. The four primary trials (16–19), met the operational criteria for driving pressure-guided ventilation, but the pooled intervention is mechanistically heterogeneous: three trials used PEEP titration and one used tidal-volume titration. The pooled estimate should therefore be interpreted as an average effect across a mechanistically heterogeneous intervention class rather than as a single homogeneous strategy. Exploratory supportive analyses—including a fixed-effect model (RR 0.61, *p* = 0.007), the pooled absolute risk difference (RD: −7.6%, *p* = 0.027), and an exploratory broadened sensitivity analysis incorporating two additional trials of individualised PEEP strategies (20, 21) (RR: 0.70; 95% CI: 0.42–1.15)—were directionally concordant but yielded statistically discrepant conclusions. We interpret this pattern as an indicator of evidence fragility rather than as independent confirmation of effect.

Intervention fidelity was well preserved across the four primary trials (16–19), as achieved driving pressure was consistently lower in the intervention groups, with between-group separations of approximately 1–3 cmH₂O during one-lung ventilation. Intraoperative oxygenation tended to be numerically higher in the driving pressure-guided groups, and dynamic compliance improved where reported. By contrast, hospital and ICU length of stay, mortality, and intraoperative vasopressor use did not show clear or consistent between-group differences. The prespecified co-primary outcome of clinically significant intraoperative haemodynamic adverse events could not be assessed as planned because none of the included trials reported it as a defined adverse event; as documented in the Methods, this represents a deviation from the registered PROSPERO protocol, and only one of the two originally registered co-primary outcomes could ultimately be assessed. One trial reported lower bronchoalveolar lavage fluid interleukin-6 levels in the intervention group (19), suggesting a possible reduction in intraoperative inflammatory signalling, although this finding remains preliminary.

Despite the directionally favourable point estimate across three of the four trials (16–18) (individual risk ratios 0.30 to 0.56), the primary meta-analysis did not yield a statistically significant result. The non-significant primary estimate should be interpreted in light of the limited number of trials, patients, and events (four trials, 654 patients, 100 events), which restricts precision; with so few trials, the confidence interval is necessarily wide, and a clinically important benefit can be neither confirmed nor excluded. Beyond imprecision, two additional factors likely contributed to between-study variability. First, the operational definition of PPCs and the postoperative ascertainment window varied markedly across the four primary trials—from Melbourne Group Scale ≥4 at 3 days (16, 17) to an ECCG-defined composite at 7 days (18) and study-specific composites (19). This outcome-level heterogeneity reflects the absence of a universally adopted PPC definition in thoracic anaesthesia and may itself account for a substantial portion of the between-study statistical heterogeneity (*I*^2^ = 49.7%), independent of any true variation in intervention effect (34). The pooled effect estimate should therefore be interpreted as an average across heterogeneous outcome constructs, not as an estimate of effect on a single standardised PPC endpoint. Second, Yan et al. (19) was the main contributor to between-study inconsistency, showing no apparent effect (RR: 1.03) in a trial that used tidal-volume titration rather than PEEP titration to achieve the driving pressure target; PPC rates were also high in both groups (approximately 39%).

The discrepancy between the non-significant relative effect estimate and the statistically significant absolute risk difference deserves careful and restrained interpretation. Rather than being viewed as convergent support for an intervention effect, the discrepancy is better understood as an indicator of evidence fragility: the two scales yield statistically different conclusions because the underlying evidence is small, heterogeneous in baseline risk, and imprecise. In meta-analyses with few studies, substantial variation in baseline event rates, and conservative variance adjustments such as Hartung-Knapp (35), relative and absolute scales can diverge. The absolute metric is more heavily influenced by trials with higher event rates, which contribute disproportionately to the pooled RD estimate. Because the primary relative effect estimate crossed the null and remained imprecise, we do not interpret the statistically significant RD as independent confirmation of a treatment effect. The primary random-effects analysis—not the absolute risk difference—remains the appropriate basis for interpretation.

The physiological rationale for driving pressure-guided ventilation during one-lung ventilation rests on the relationship between tidal volume, functional lung size, and pulmonary strain (5, 36). Driving pressure reflects the ratio of tidal volume to respiratory system compliance and therefore captures the degree of mechanical stretch imposed on aerated lung tissue. During one-lung ventilation, exclusion of one lung substantially reduces the available ventilating volume and functional residual capacity, concentrating mechanical forces on the dependent lung (1, 7, 37). Under these conditions, a tidal volume that might be considered protective during two-lung ventilation can generate disproportionately high driving pressures and potentially exceed the threshold for ventilator-induced lung injury. Accordingly, targeting driving pressure during one-lung ventilation has a strong physiological rationale beyond the use of fixed tidal-volume or fixed PEEP settings alone (5, 6).

A descriptive observation from this review is that the three primary trials showing point estimates favouring reduced postoperative pulmonary complications (16–18) all used PEEP titration to minimise driving pressure, whereas the single trial showing no apparent effect (19) used tidal-volume titration to maintain driving pressure within a predefined target range. We emphasise that this pattern is strictly hypothesis-generating and does not constitute evidence of differential efficacy between implementation pathways. Formal interaction testing was not feasible with only four trials and one Vt-titration study, and the apparent pattern could equally reflect confounding by trial-level characteristics unrelated to the implementation pathway—such as different patient populations, baseline PPC rates, or PPC definitions—rather than a true pathway effect. With this important caveat, we note the physiological speculation that PEEP titration to the lowest driving pressure may simultaneously optimise end-expiratory lung volume and alveolar recruitment, whereas tidal-volume reduction alone may lower driving pressure without necessarily improving ventilation distribution or limiting atelectatic collapse. This hypothesis remains speculative and will require direct comparative testing in adequately powered head-to-head trials.

The present review addresses a more focused clinical question than most previous syntheses of intraoperative ventilation during one-lung ventilation (8, 38). Earlier systematic reviews and trials in this field have predominantly evaluated broader protective ventilation bundles, individualised PEEP strategies, or open-lung approaches without isolating driving pressure as the explicit guiding target. By restricting the primary analysis to trials in which driving pressure minimisation or threshold attainment was the stated principal decision rule for ventilator adjustment, this review provides a more specific assessment of driving pressure-guided ventilation as an intervention construct, rather than of protective ventilation as a broader composite strategy. The eight additional randomized trials identified during the review process (26–33) illustrate this distinction: some evaluated individualised PEEP titration using compliance-guided or recruitment manoeuvre-based decrement protocols (26, 28), others tested broader perioperative protective ventilation bundles such as the multicentre iPROVE-OLV trial (27), and still others focused primarily on physiological endpoints, including variable ventilation (33) or biventricular function during oesophageal surgery (31). Collectively, these studies support the broader concept that individualised ventilatory management can improve intraoperative physiology during one-lung ventilation (29, 30, 32), but because their intervention constructs differed from an explicit driving pressure-guided comparison, they were treated as contextual and hypothesis-supporting evidence. The supplementary observational evidence should likewise be interpreted in context. Two adjusted cohort studies (24, 25) found that higher intraoperative driving pressure was independently associated with greater PPC risk, which supports the biological plausibility of the randomized findings. However, two other observational studies (22, 23) did not confirm this association, and differences in exposure definition, covariate adjustment, and outcome ascertainment limit direct comparability across cohorts. Exposure–outcome associations derived from observational data cannot substitute for the causal inference provided by randomized strategy comparisons; the observational evidence therefore complements the randomized findings by supporting biological plausibility, but does not replace strategy-based causal evidence from trials.

This review has several methodological strengths. It addressed a narrowly defined clinical question—driving pressure-guided ventilation during one-lung ventilation for thoracic surgery—thereby avoiding conflation with broader and more heterogeneous protective ventilation strategies. The strict primary analysis included only trials in which driving pressure was explicitly stated as the principal ventilatory target (16–19), while trials with indirect or incidental driving pressure lowering were handled separately in a broadened sensitivity analysis (20, 21). Observational evidence (22–25) was synthesised narratively rather than pooled with the randomized data, preserving the distinction between strategy-based causal evidence and exposure–outcome associations. Multiple sensitivity analyses, including fixed-effect, odds ratio, risk difference, and broadened eligibility approaches, were conducted to examine the robustness of the findings. The certainty of evidence was formally assessed using the GRADE framework (15).

Several limitations should also be acknowledged. The primary analysis included only four single-centre randomized trials (16–19), all conducted in East Asia (three in China, one in South Korea), contributing a total of 654 patients and 100 PPC events. This small and geographically concentrated evidence base limited statistical power, precluded formal subgroup interaction testing, made formal assessment of publication bias infeasible, and constrains the generalisability of findings to other healthcare systems, perioperative practices, and patient populations. PPC definitions and assessment windows varied across trials (34), which may have contributed to between-study heterogeneity independent of the intervention itself, and we could not resolve this through post-hoc harmonisation because component-level PPC data were not consistently reported. The four primary trials used two mechanistically distinct implementation pathways for driving pressure reduction (PEEP titration vs. tidal-volume titration); although all four satisfied the operational criteria for explicit driving pressure-guided ventilation, treating them as a single intervention class for quantitative synthesis was a methodological compromise, necessary given the limited evidence base but introducing clinical heterogeneity that cannot be resolved with the available data. The observation that the three PEEP-titration trials favoured the intervention whereas the single Vt-titration trial did not is presented as hypothesis-generating only, because formal interaction testing was not feasible and the pattern could reflect trial-level confounders unrelated to the implementation pathway. One of the two registered co-primary outcomes—clinically significant intraoperative haemodynamic adverse events—could not be assessed as originally intended because no trial reported it as a defined adverse event, and the available vasopressor data were too heterogeneous for quantitative synthesis; this deviation from the registered protocol is documented in the Methods. Of the six prespecified subgroup analyses and five prespecified sensitivity analyses (in addition to the broadened eligibility extension), only two sensitivity analyses and one extension were quantitatively estimable, primarily because of the limited number of included trials; this substantial gap between planned and actually estimable analyses is itself a methodological limitation and is detailed in the Supplementary material. The two trials included in the exploratory broadened sensitivity analysis (20, 21) were not strictly driving pressure-guided, and their inclusion broadened the intervention definition beyond the strict primary eligibility framework. The observational studies (22–25) were also subject to residual confounding and heterogeneity in exposure measurement.

The current evidence is not sufficient to recommend driving pressure-guided ventilation as a universal standard of care during one-lung ventilation. However, the directionally favourable point estimate across most included trials, combined with physiological rationale (5, 6, 36) and supplementary observational evidence (24, 25), suggests that monitoring driving pressure as one component of individualised ventilatory decision-making is a reasonable approach, particularly when interpreted alongside other physiological parameters. In clinical practice, anaesthesiologists managing one-lung ventilation may consider incorporating driving pressure monitoring into individualised ventilator adjustment, especially in patients at higher baseline risk of postoperative pulmonary complications (2, 3, 39).

Several priorities for future research emerge from the current evidence base. First, large, adequately powered, multicentre, and geographically diverse randomized trials with postoperative pulmonary complications as the primary endpoint are needed, both to confirm or refute the signal observed in this review and to improve the generalisability of findings beyond single-centre East Asian populations. Second, harmonised PPC definitions—ideally aligned with established consensus frameworks such as the EPCO definitions (34)—would facilitate more reliable cross-trial comparisons and reduce outcome-level heterogeneity, which was a major source of between-study variability in the current review. Third, future trials should prespecify and systematically report intraoperative haemodynamic safety endpoints, including clearly defined vasopressor requirements, to address the safety gap identified in this review. Fourth, head-to-head comparisons of different driving pressure implementation pathways—specifically PEEP-titration-based versus tidal-volume-titration-based approaches—would help clarify whether the mechanism of driving pressure reduction influences clinical outcomes; the current review did not provide evidence of differential efficacy between these pathways, and the apparent descriptive difference should be regarded as a hypothesis requiring prospective testing rather than as an established finding. Fifth, reporting the achieved between-group driving pressure separation should become standard practice, because it directly determines intervention fidelity and interpretability. Finally, identifying patient subgroups most likely to benefit—such as those with pre-existing pulmonary disease, reduced lung compliance, or higher baseline PPC risk—would support more targeted application of driving pressure-guided strategies (39). Together, these advances would help determine not only whether driving pressure-guided ventilation is effective, but also how, in whom, and by which implementation pathway it should be applied during one-lung ventilation.

## Conclusion

In this systematic review and meta-analysis, the primary random-effects analysis of four randomized controlled trials did not demonstrate a statistically significant reduction in postoperative pulmonary complications with driving pressure-guided ventilation during one-lung ventilation for thoracic surgery (RR: 0.60; 95% CI: 0.26–1.35; *p* = 0.14). The overall certainty of evidence was low, owing to inconsistency and imprecision. Exploratory supportive analyses were directionally concordant but should not be interpreted as independent confirmation of effect. The available evidence supports the physiological plausibility of driving pressure-guided ventilation as a lung-protective strategy during one-lung ventilation, but remains insufficient to establish a definitive treatment effect. The current data are therefore hypothesis-generating. Adequately powered, multicentre, and geographically diverse randomized trials with harmonised outcome definitions are needed to determine whether this approach improves postoperative outcomes and should be adopted more broadly in thoracic anaesthesia.

## Data Availability

The original contributions presented in the study are included in the article/Supplementary material, further inquiries can be directed to the corresponding author.
