# Supplementary material for: Driving pressure-guided ventilation during one-lung ventilation for thoracic surgery: a systematic review and meta-analysis
Source: Front Med (Lausanne). 2026 May 20;13:1837064. doi: 10.3389/fmed.2026.1837064 (PMC13229865; doi:10.3389/fmed.2026.1837064)
Supplement: Supplementary file 1 [file Data_Sheet_1.PDF]

Supplementary Appendix S1. Full electronic search strategies for all databases

Search strategies were developed for MEDLINE (PubMed) and adapted for each additional database. No restrictions were applied regarding language, country, or publication date. All searches were conducted on 19 March 2026.

MEDLINE (via PubMed) Search date: 19 March 2026 Hits: 25

("one-lung ventilation"[MeSH] OR "one lung ventilation"[TIAB] OR "single lung ventilation"[TIAB] OR "OLV"[TIAB]) AND ("driving pressure"[TIAB] OR "delta P"[TIAB] OR "transpulmonary pressure"[TIAB] OR "plateau pressure"[TIAB] OR "Pplat"[TIAB] OR "respiratory system compliance"[TIAB]) AND ("thoracic surgery"[MeSH] OR "lung resection"[TIAB] OR "lobectomy"[TIAB] OR "esophagectomy"[TIAB] OR "oesophagectomy"[TIAB] OR "VATS"[TIAB] OR "video-assisted thorac*"[TIAB]) AND (random*[TIAB] OR trial[TIAB] OR cohort[TIAB] OR observational[TIAB])

Embase (via Embase.com) Search date: 19 March 2026 Hits: 75

('one lung ventilation'/exp OR 'one lung ventilation':ti,ab OR 'single lung ventilation':ti,ab OR OLV:ti,ab) AND ('driving pressure':ti,ab OR 'delta P':ti,ab OR 'transpulmonary pressure':ti,ab OR 'plateau pressure':ti,ab OR pplat:ti,ab OR 'respiratory system compliance':ti,ab) AND ('thoracic surgery'/exp OR 'lung resection':ti,ab OR lobectomy:ti,ab OR esophagectomy:ti,ab OR oesophagectomy:ti,ab OR VATS:ti,ab OR 'video-assisted thorac*':ti,ab) AND (random*:ti,ab OR trial:ti,ab OR cohort:ti,ab OR observational:ti,ab)

Cochrane Central Register of Controlled Trials (CENTRAL) Search date: 19 March 2026 Hits: 71

("one lung ventilation" OR "single lung ventilation" OR OLV) AND ("driving pressure" OR "delta P" OR "transpulmonary pressure" OR "plateau pressure" OR Pplat OR "respiratory system compliance") AND ("thoracic surgery" OR "lung resection" OR lobectomy OR esophagectomy OR oesophagectomy OR VATS OR "video-assisted thorac*")

Web of Science Core Collection Search date: 19 March 2026 Hits: 46

TS=("one lung ventilation" OR "single lung ventilation" OR OLV) AND TS=("driving pressure" OR "delta P" OR "transpulmonary pressure" OR "plateau pressure" OR Pplat OR "respiratory system compliance") AND TS=("thoracic surgery" OR "lung resection" OR lobectomy OR esophagectomy OR oesophagectomy OR VATS OR "video-assisted thorac*") AND TS=(random* OR trial OR cohort OR observational)

Scopus Search date: 19 March 2026 Hits: 52

TITLE-ABS-KEY("one lung ventilation" OR "single lung ventilation" OR OLV) AND TITLE-ABS-KEY("driving pressure" OR "delta P" OR "transpulmonary pressure" OR "plateau pressure" OR Pplat OR "respiratory system compliance") AND TITLE-ABS-KEY("thoracic surgery" OR "lung resection" OR lobectomy OR esophagectomy OR oesophagectomy OR VATS OR "video-assisted thorac*") AND TITLE-ABS-KEY(random* OR trial OR cohort OR observational)

ClinicalTrials.gov Search date: 19 March 2026 Hits: 6

("one lung ventilation" OR OLV) AND ("driving pressure" OR "plateau pressure")

WHO International Clinical Trials Registry Platform (ICTRP) Search date: 19 March 2026 Hits: 7

Advanced Search: Title: ("one lung ventilation" OR OLV OR "single lung ventilation") Intervention: ("driving pressure" OR "plateau pressure" OR Pplat)

Supplementary Figure S1. Forest plot of postoperative pulmonary complications in the broadened sensitivity analysis including six randomized controlled trials

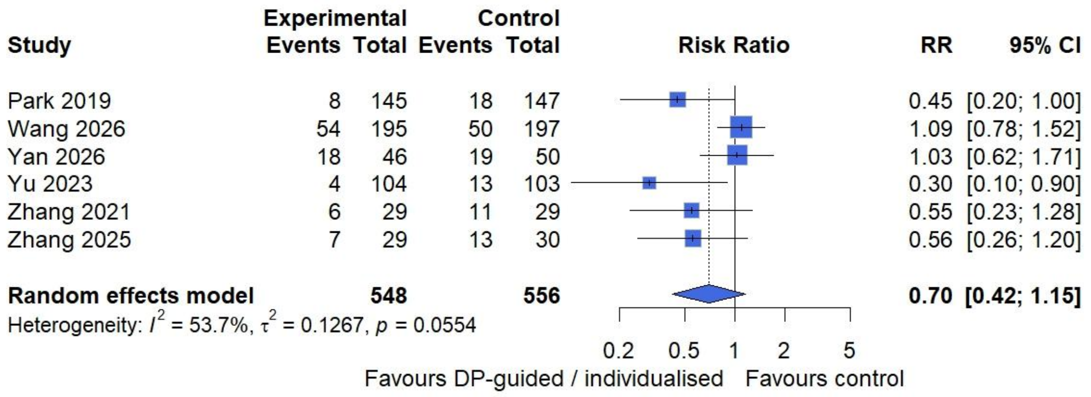

The four primary trials of explicit driving pressure-guided ventilation are shown alongside two additional trials of individualised PEEP titration strategies in which lower driving pressure was a documented physiological consequence rather than the prespecified decision target (Zhang 2021, Wang 2026). Meta-analysis was performed using a random-effects model with restricted maximum likelihood estimation and Hartung-Knapp adjustment.

Supplementary Table S1. Verbatim intervention statements, classification outcomes, and rationale for all randomized controlled trials assessed against the driving pressure-guided ventilation classification criteria

Classification criteria (from Methods, Eligibility criteria, Intervention subsection): Trials were classified as explicitly driving pressure-guided if they satisfied all three of the following pre-specified criteria:

Criterion (i): The study protocol or published report explicitly named driving pressure (or ΔP) as the primary ventilatory target or primary decision rule for ventilator adjustment during OLV, with a verbatim statement extractable from the Methods section of the original report.

Criterion (ii): The protocol specified either a driving pressure threshold (e.g., ΔP ≤14 or ≤15 cmH_2O) or a driving pressure minimisation procedure (e.g., stepwise titration to the lowest ΔP) as the operational decision rule for ventilator setting adjustment.

Criterion (iii): The intervention protocol did not additionally specify an alternative primary target (such as oxygenation, compliance maximisation, or recruitable lung volume) that would take precedence over the driving pressure target.

Classification outcomes:

Study	Verbatim intervention statement (extracted from the original report)	Operational target	Criterion (i)	Criterion (ii)	Criterion (iii)	Classification	Rationale

Study	Verbatim intervention statement (extracted from the original report)	Operational target	Criterion (i)	Criterion (ii)	Criterion (iii)	Classification	Rationale
**Park 2019**	"For driving pressure-guided ventilation, patients received the same FIO_2 and V_T, but individualized lowest driving pressure was applied during one-lung ventilation... Then the PEEP level that produced the lowest driving pressure was chosen and maintained throughout one-lung ventilation."	PEEP titration to minimise ΔP	✓	✓	✓	Primary (strict DP-guided)	Driving pressure minimisation was explicitly stated as the principal decision rule for ventilator adjustment. PEEP was the operational lever, but the target was ΔP.
**Yu 2023**	"For group D, patient received individualised PEEP based on the lowest driving pressure during OLV... Afterwards, the PEEP level which delivered the lowest ΔP was determined and maintained for the duration of OLV period."	PEEP titration to minimise ΔP	✓	✓	✓	Primary (strict DP-guided)	Driving pressure minimisation was explicitly stated as the principal decision rule. PEEP titration was the mechanism; ΔP minimisation was the target.
**Zhang 2025**	"PEEP was decreased stepwise fashion from 10 to 0 cmH_2O... The PEEP associated with the lowest DP was identified as optimal, and remained the PEEP setting for the remainder of that procedural interval."	PEEP titration to minimise ΔP	✓	✓	✓	Primary (strict DP-guided)	Decremental PEEP trial was used as the procedural method, but the explicit target was the lowest ΔP.
**Yan 2026**	"In the driving pressure-guided VT group, tidal volume was adjusted to	Vt titration to maintain ΔP within	✓	✓	✓	Primary (strict DP-guided)	Tidal volume was the operational lever, but the

Study	Verbatim intervention statement (extracted from the original report)	Operational target	Criterion (i)	Criterion (ii)	Criterion (iii)	Classification	Rationale
	maintain a driving pressure of 8 to 10 cm H_2O during OLV."	threshold range					target was a ΔP threshold range. This is the only primary trial using a Vt-driven rather than PEEP-driven pathway.
**Zhang 2021**	"Next, group A was titrated using a PEEP decremental trial... until the maximum pulmonary compliance (C_{PAT}) was obtained at the optimum PEEP level. Then the PEEP application level of Group A was changed to the optimal PEEP."	PEEP titration to maximum compliance	✗	✗	✗	Broadened sensitivity	The explicit target was maximum compliance, not ΔP. Lower ΔP was observed as a downstream physiological consequence. Fails Criterion (i).
**Wang 2026**	"Positive end-expiratory pressure was titrated using electrical impedance tomography... Optimal PEEP was based on the intersection of the lung overdistension and reversible atelectasis curves."	EIT-guided PEEP titration	✗	✗	✗	Broadened sensitivity	The explicit target was EIT-guided optimisation of regional ventilation distribution, not ΔP. Fails Criterion (i).
**Ferrando 2014**	*Paraphrased:* Individualized PEEP was set by a PEEP decrement trial after a recruitment maneuver.	Recruitment manoeuvre + PEEP decrement for oxygenation and mechanics	✗	✗	✗	Narrative only	Primary target was recruitment-based open-lung strategy, not ΔP. Reported intraoperative oxygenation and mechanics, not PPCs as comparative

Study	Verbatim intervention statement (extracted from the original report)	Operational target	Criterion (i)	Criterion (ii)	Criterion (iii)	Classification	Rationale
							intervention.
**Ferrando 2018**	*Paraphrased:* The individualized open-lung approach combined an alveolar recruitment maneuver followed by individualized PEEP set to the lowest dynamic compliance driving pressure.	Multi-component open-lung bundle	✗	✗	✗	Narrative only	Intervention was a composite perioperative open-lung bundle rather than a strictly DP-guided strategy. Fails Criterion (iii) due to multi-component intervention construct.
**Xu 2021**	*Paraphrased:* Individualized PEEP titration strategies (static compliance-based and oxygen saturation-based) during one-lung ventilation.	Compliance-based or SpO_2-based PEEP titration	✗	✗	✗	Narrative only	Explicit targets were static compliance or oxygen saturation, not ΔP. Fails Criterion (i).
**Licker 2009**	*Paraphrased:* A lung-protective ventilation strategy including low tidal volume, PEEP, and recruitment manoeuvres.	Composite protective ventilation bundle	✗	✗	✗	Narrative only	Multi-component protective ventilation bundle without ΔP as explicit primary target.
**Marret 2018**	*Paraphrased:* Protective ventilation (low tidal volume + PEEP + recruitment manoeuvres) versus conventional ventilation.	Composite protective ventilation bundle	✗	✗	✗	Narrative only	Composite protective ventilation bundle; ΔP was not the explicit decision rule.

Study	Verbatim intervention statement (extracted from the original report)	Operational target	Criterion (i)	Criterion (ii)	Criterion (iii)	Classification	Rationale
**Li 2022**	*Paraphrased:* Comparison of different PEEP levels during one-lung ventilation under a lower FiO_2.	Fixed PEEP level comparison	✗	✗	✗	Narrative only	Compared fixed PEEP levels; primary outcomes were oxygenation and biventricular function, not ΔP-guided strategy.
**Piccioni 2023**	*Paraphrased:* Protective ventilation strategy during one-lung ventilation for major lung resection.	Composite protective ventilation strategy	✗	✗	✗	Narrative only	Broader protective ventilation bundle, not explicitly ΔP-guided.
**Wittenstein 2024**	*Paraphrased:* Variable versus conventional volume-controlled one-lung ventilation.	Variable tidal volume delivery	✗	✗	✗	Narrative only	Primary intervention construct was variable vs conventional Vt delivery; physiological endpoints were gas exchange and respiratory mechanics, not ΔP-guided comparison.

Notes: Verbatim intervention statements were extracted directly from the Methods sections of the original reports for the primary and broadened sensitivity trials. Where trials were designated as contextual narrative-only references, paraphrased statements reflecting the core intervention are provided. Classification was performed independently by two reviewers, with disagreements resolved through discussion and third-reviewer adjudication where needed. "Primary (strict DP-guided)" trials were included in the primary quantitative synthesis (k = 4). "Broadened sensitivity" trials (k = 2) were included only in the prespecified broadened sensitivity analysis, where their inclusion extended the intervention definition beyond the strict DP-guided framework. "Narrative only" trials (k = 8) were retained for Discussion context but did not contribute to any quantitative synthesis.

Supplementary Table S2. Detailed risk of bias assessment for included randomized controlled trials using the Cochrane Risk of Bias 2 (RoB 2) tool

Study	D1: Randomisation process	D2: Deviations from intended interventions	D3: Missing outcome data	D4: Measurement of the outcome	D5: Selection of the reported result	Overall Risk of Bias
**Park 2019**	**Low risk.** Computer-generated random sequence and opaque sealed envelopes were used; baseline characteristics were balanced.	**Some concerns.** Anaesthesiologists could not be blinded to the assigned ventilator settings. However, ITT analysis was used, and deviations were unlikely to selectively impact outcomes.	**Low risk.** No missing data; 292 out of 292 randomized patients were successfully followed up and analyzed.	**Low risk.** Postoperative pulmonary complications (PPCs) were evaluated by independent assessors blinded to group allocation.	**Low risk.** The reported primary and secondary outcomes correspond perfectly with the pre-registered trial protocol.	**Some concerns**
**Yu 2023**	**Low risk.** Randomization was performed using a computer-generated list and concealed via sealed opaque envelopes.	**Some concerns.** Non-blinded anaesthesiologists administered the intervention. Analysis was performed according to the ITT principle.	**Low risk.** Complete follow-up was achieved for all 207 analyzed participants with no missing outcome data.	**Low risk.** A blinded independent observer collected postoperative clinical data and assessed PPCs.	**Low risk.** Results match the prespecified analysis plan registered prior to trial completion.	**Some concerns**
**Zhang 2025**	**Low risk.** SPSS-generated random numbers and sealed opaque envelopes ensured adequate sequence generation and allocation concealment.	**Some concerns.** The nature of the intervention precluded blinding of the attending anaesthesiologists, though strict protocol adherence was reported.	**Low risk.** Only 1 patient dropped out (due to a change in surgical approach), leaving 59/60 patients analyzed (>98% completeness).	**Low risk.** PPCs were assessed according to objective ECCG criteria by blinded personnel.	**Low risk.** No evidence of selective reporting; outcomes align with the registered trial endpoints.	**Some concerns**
**Yan**	**Low risk.**	**Some concerns.** Lack of	**Low risk.** Complete	**Low risk.** Blinded	**Low risk.** All	**Some**

Study	D1: Randomisation process	D2: Deviations from intended interventions	D3: Missing outcome data	D4: Measurement of the outcome	D5: Selection of the reported result	Overall Risk of Bias
**2026**	Computer-generated randomization and opaque sealed envelopes were appropriately implemented.	blinding for the clinicians adjusting the ventilator, balanced by an appropriate ITT analytical framework.	outcome data were available for all 96 enrolled patients.	assessors evaluated postoperative outcomes, and respiratory mechanics were derived from objective ventilator data.	prespecified outcomes in the trial registry were reported in the final manuscript.	**concerns**
**Zhang 2021**	**Low risk.** A random number table and sealed envelopes were used; groups were comparable at baseline.	**Some concerns.** Anaesthesiologists were aware of the PEEP assignments. The effect of assignment to intervention was analyzed.	**Low risk.** Outcome data were completely analyzed for all 58 randomized participants without attrition.	**Low risk.** Independent outcome assessors were explicitly blinded to the intraoperative ventilatory management.	**Low risk.** Published outcomes are consistent with the registered primary endpoints.	**Some concerns**
**Wang 2026**	**Low risk.** Computer-generated sequences and opaque envelopes provided adequate randomization and allocation concealment.	**Some concerns.** Blinding of the anesthesia team was impossible due to EIT and PEEP adjustments, but an ITT approach was strictly maintained.	**Low risk.** A very low dropout rate (~3%) with balanced missingness between groups; unlikely to introduce bias.	**Low risk.** PPCs were determined by researchers blinded to the randomization group using standard diagnostic criteria.	**Low risk.** The manuscript reports all outcomes as defined in the prospective clinical trial registry.	**Some concerns**

Each domain was judged as "low risk", "some concerns", or "high risk" according to the RoB 2 signalling questions and algorithm. The overall judgement was derived following the RoB 2 decision rules. Studies marked with an asterisk (*) in the main text (Zhang 2021, Wang 2026) were included in the broadened sensitivity analysis only.

Supplementary Table S3. Detailed risk of bias assessment for supplementary observational studies using the Risk Of Bias In Non-randomised Studies of Interventions (ROBINS-I) tool

Study	D1: Confounding	D2: Selection of participants	D3: Classification of interventions	D4: Deviations from intended interventions	D5: Missing data	D6: Measurement of outcomes	D7: Selection of reported result	Overall Risk of Bias
**Cirenei 2026**	**Moderate.** Adjusted for age, neoadjuvant therapy, and surgery duration, but lacked adjustment for BMI and specific baseline pulmonary function.	**Moderate.** Retrospective cohort design; selection of analysed patients depended on complete electronic health records availability.	**Low.** Driving pressure was objectively calculated as a time-weighted average directly from ventilator data.	**Moderate.** Co-interventions (e.g., perioperative fluid or pain management) might vary and could not be fully controlled.	**Low.** The authors appropriately used multiple imputation methods to address missing clinical data.	**Moderate.** Retrospective ascertainment of PPCs from medical charts without confirmed assessor blinding to DP exposure.	**Moderate.** Retrospective analysis without a prospectively published study protocol or analysis plan.	**Moderate risk**
**Yang 2025**	**Moderate.** Multivariable models included age, ASA, and OLV duration, but missed key specific variables like BMI or FEV1.	**Moderate.** Retrospective inclusion; excluded patients with missing intraoperative ventilator data, potentially introducing mild selection bias.	**Low.** DP exposure was accurately extracted as the time-weighted median value from the automated anaesthesia record system.	**Moderate.** Potential for unbalanced perioperative care standards over the study period across different DP strata.	**Moderate.** Patients with missing data were excluded rather than imputed, though the proportion was relatively small.	**Moderate.** Postoperative complications were extracted retrospectively from registries; assessor blinding is inherently lacking.	**Moderate.** No pre-registered protocol available to verify the predefined analysis plan.	**Moderate risk**

**Okahara 2018**	**Moderate.** Adjusted for BMI and FEV1, but only adjusted for total surgery duration rather than the specific duration of OLV.	**Low.** Prospective, consecutive enrolment of eligible patients without selection based on exposure or outcome.	**Low.** DP was prospectively and explicitly defined and measured during the first 2 hours of OLV.	**Moderate.** Postoperative care pathways might vary, introducing potential co-intervention bias outside the study's control.	**Low.** Prospective design ensured high data completeness with minimal missing covariate or outcome data.	**Moderate.** It was not explicitly stated whether the outcome assessors were blinded to the intraoperative DP levels.	**Low.** The analysis aligned with the prospective observational study design.	**Moderate risk**
**Uhlig 2020**	**Serious.** The specific thoracic subgroup analysis used Cox-proportional hazard models *without* adjustment for covariates, leaving critical confounding unaddressed.	**Low.** Data were drawn from a large, prospective, consecutive multinational cohort (LAS VEGAS study).	**Moderate.** Evaluated the intraoperative mean DP across both OLV and TLV phases, lacking specific isolation of the OLV period.	**Moderate.** Given the multinational design, background perioperative care varied widely and was not standardized.	**Low.** Excellent data completeness due to the rigorous prospective nature of the parent trial.	**Moderate.** Assessors were not explicitly blinded to the intraoperative ventilation settings.	**Moderate.** The specific thoracic subgroup analysis was likely a post-hoc exploration rather than a primary registered endpoint.	**Serious risk**

Each domain was judged as "low", "moderate", "serious", or "critical" risk of bias according to the ROBINS-I framework. The overall judgement corresponds to the most severe domain-level judgement for each study. These observational studies were synthesised narratively and were not pooled quantitatively with the randomized trials.

Supplementary Table S4. Post-hoc sensitivity risk of bias assessment for supplementary observational studies using the Quality In Prognosis Studies (QUIPS) tool

Study	D1: Study participation	D2: Study attrition	D3: Prognostic factor measurement	D4: Outcome measurement	D5: Study confounding	D6: Statistical analysis and reporting	Overall Risk of Bias
**Okahara 2018**	**Low.** Prospective, multicentre design with consecutive enrolment and clear inclusion/exclusion criteria.	**Low.** Prospective design with complete follow-up for the short-term postoperative observation window.	**Low.** DP was prospectively and standardisedly measured during the first 2 hours of OLV.	**Low.** Postoperative pulmonary complications (PPCs) were defined using standard clinical criteria.	**Moderate.** Adjusted for key confounders (age, BMI, FEV1, surgery duration), but did not specifically adjust for the duration of one-lung ventilation (OLV).	**Low.** Multivariable logistic regression was appropriately conducted and completely reported.	**Moderate risk**
**Uhlig 2020**	**Low.** Data drawn from a large, prospective, consecutive multinational cohort (LAS VEGAS study).	**Low.** High data completeness due to the rigorous prospective nature of the parent trial.	**Moderate.** Evaluated intraoperative mean DP across both OLV and TLV phases, lacking specific isolation of the OLV period.	**Low.** Standardised PPC composite outcome captured up to postoperative day 5.	**High.** The specific thoracic subgroup analysis explicitly used unadjusted models, leaving critical confounders completely unaddressed.	**Moderate.** Subgroup analysis limitations; lack of adjusted effect estimates for the exposure of interest.	**High risk**
**Yang 2025**	**Low.** Large consecutive	**Low.**	**Low.** DP exposure	**Low.** Pre-defined	**Moderate.**	**Low.** Statistical	**Moderate**

	cohort of eligible surgical patients.	Retrospective electronic medical record data extraction with high completeness.	was accurately extracted as the time-weighted median value from the automated anaesthesia record system.	clinical criteria used for PPC identification.	Multivariable models included age, ASA, and OLV duration, but missed specific quantitative baseline pulmonary function (e.g., FEV1) and BMI.	methods were appropriate and findings completely reported.	**risk**
**Cirenei 2026**	**Low.** Consecutive cohort of oesophagectomy patients with clear inclusion criteria.	**Low.** The authors appropriately used multiple imputation methods to address missing clinical data, minimising attrition bias.	**Low.** DP was objectively calculated as a time-weighted average directly from ventilator data.	**Low.** PPCs were assessed using standard clinical grading systems.	**Moderate.** Adjusted for age, neoadjuvant therapy, and surgery duration, but lacked specific adjustment for BMI and baseline pulmonary function.	**Low.** Statistical methods (including multiple imputation) were appropriate and well reported.	**Moderate risk**

Notes: Each domain was judged as "low", "moderate", or "high" risk of bias according to the QUIPS framework. The overall judgement was determined primarily by the most consequential domain (typically Study confounding, D5) in the context of the overall methodological quality. This post-hoc sensitivity appraisal was performed in response to peer review to complement the pre-specified ROBINS-I assessment. The overall judgements from both frameworks were broadly concordant: three studies (Okahara 2018, Yang 2025, Cirenei 2026) were rated as moderate risk of bias overall, and one study (Uhlig 2020) was rated as the highest category of bias available within each tool, with study confounding being the predominant concern in all four cases.

Supplementary Table S5. Prespecified subgroup and sensitivity analyses: planned versus actually estimable

Analysis type	Prespecified in PROSPERO (CRD420261329253)	Actually performed	Rationale if not feasible / Location reported
**Subgroup analyses (primary RCT layer)**			
DP-guided strategy type (PEEP-driven vs Vt-driven vs combined)	Yes	Descriptively only (no formal interaction test)	Only 4 trials; 3 PEEP-driven, 1 Vt-driven; insufficient studies per subgroup for formal quantitative interaction testing.
DP target threshold (≤14 vs ≤15 vs minimisation)	Yes	Not feasible	Only 4 trials; heterogeneous thresholds do not permit grouping for formal analysis.
Recruitment manoeuvre use (routine RM vs no routine RM)	Yes	Not feasible	Too few trials per subgroup.
Surgical category (lung resection vs oesophagectomy vs mixed)	Yes	Not feasible	Only 4 trials; single oesophagectomy trial and single mixed-population trial.
Background Vt strategy (≤6 vs >6 mL/kg PBW)	Yes	Not feasible	Between-trial variation insufficient for formal grouping.
High-risk lung populations (COPD/reduced FEV_1)	Yes	Not feasible	Insufficient reporting of high-risk subgroups across trials.
**Sensitivity analyses (primary RCT layer)**			

Analysis type	Prespecified in PROSPERO (CRD420261329253)	Actually performed	Rationale if not feasible / Location reported
Exclusion of RCTs at overall high risk of bias	Yes	Not applicable	No trial was judged at overall high risk of bias.
Fixed-effect (inverse-variance) model	Yes	**Performed**	Reported in Results Section 3.4.3.
Odds ratios instead of risk ratios	Yes	**Performed**	Reported in Results Section 3.4.3.
Restriction to trials with achieved ΔP separation ≥3 cmH_2O	Yes	Not feasible	Only 1 of 4 trials met this threshold (Yu 2023); single-study analysis is uninformative.
Exclusion of predominantly PEEP-driven strategies	Yes	Not feasible	Only 1 Vt-driven trial remains (Yan 2026); single-study analysis is uninformative.
Broadened eligibility (individualised PEEP with documented lower ΔP)	Yes	**Performed**	Reported in Results Section 3.4.2 as an exploratory extension.

Supplementary Table S5. Prespecified subgroup and sensitivity analyses: planned versus actually estimable

This table presents a complete mapping of all subgroup and sensitivity analyses prespecified in the PROSPERO registration (CRD420261329253) against the analyses that were actually estimable given the final included evidence base. Each analysis is classified as performed, reported descriptively only, not feasible, or not applicable, with the rationale provided where an analysis could not be conducted as originally planned. This table is provided to enhance methodological transparency by making the distinction between planned analyses and actually estimable analyses fully explicit, in line with PRISMA 2020

guidance on reporting protocol deviations and unexecutable prespecified analyses. DP = driving pressure; PEEP = positive end-expiratory pressure; Vt = tidal volume; PBW = predicted body weight; RM = recruitment manoeuvre; FEV_1 = forced expiratory volume in 1 second; COPD = chronic obstructive pulmonary disease; RR = risk ratio; OR = odds ratio.

Supplementary Table S6. Descriptive summary of prespecified subgroup and sensitivity analyses not feasible as formal quantitative comparisons

Planned analysis	Eligible studies (k)	Feasibility	Descriptive observation
**DP strategy type:** PEEP-titration vs VT-titration	3 PEEP-titration, 1 VT-titration	Not feasible (k = 1 in one subgroup)	Three PEEP-titration trials showed RR 0.30–0.56; the single VT-titration trial (Yan 2026) showed RR 1.03
**DP target threshold:** ≤14 vs ≤15 vs minimisation	3 minimisation, 1 target range (8–10)	Not feasible (no trials used ≤14 or ≤15 as fixed threshold)	All three minimisation trials favoured intervention; the target-range trial showed no effect
**Recruitment manoeuvres:** routine RM vs no RM	4 with RM (all trials)	Not feasible (no trials without RM)	All four trials used recruitment manoeuvres; comparison not possible
**Surgical category:** lung resection vs oesophagectomy	2 lung resection, 1 oesophagectomy, 1 mixed	Not feasible (k = 1 in oesophagectomy)	Both lung resection trials and the oesophagectomy trial showed RR < 1; the mixed trial (Park 2019) also favoured intervention
**Background Vt:** ≤6 vs >6 mL/kg PBW	Variable across trials	Not feasible (Vt strategies overlapped and were not clearly dichotomisable)	Not assessable
**High-risk lung populations** (COPD/low FEV_1)	No trial reported subgroup data	Not feasible (no subgroup data available)	Not assessable
**DP separation ≥3 cmH_2O**	2 trials (Yu 2023, Zhang 2025) vs 2 trials <3	Not feasible (k = 2 per subgroup)	Two trials with ≥3 cmH_2O separation (Yu, Zhang) showed RR 0.30 and 0.56; two trials with <3 cmH_2O (Park, Yan) showed RR 0.45 and 1.03
**Exclude PEEP-driven strategy**	1 trial remaining (Yan 2026)	Not feasible (k = 1)	Single remaining trial showed no effect (RR 1.03)

Supplementary Table S3. Descriptive summary of prespecified subgroup and sensitivity analyses not feasible as formal quantitative comparisons

These subgroup and sensitivity analyses were prespecified in the protocol but could not be conducted as formal quantitative interaction tests owing to the limited number of included trials (k = 4). Descriptive observations are provided for transparency and should be interpreted with caution. DP, driving pressure; PEEP, positive end-expiratory pressure; RR, risk ratio; RM, recruitment manoeuvre; VT, tidal volume; PBW, predicted body weight.
